# γδ T cell-mediated activation of cDC1 orchestrates CD4^+^ Th1 cell priming in malaria

**DOI:** 10.3389/fimmu.2024.1426316

**Published:** 2024-08-15

**Authors:** Yarob Ibraheem, Ganchimeg Bayarsaikhan, Maria Lourdes Macalinao, Kazumi Kimura, Katsuyuki Yui, Taiki Aoshi, Shin-Ichi Inoue

**Affiliations:** ^1^ Department of Immunology, Graduate School of Biomedical Sciences, Nagasaki University, Nagasaki, Japan; ^2^ School of Tropical Medicine and Global Health, Nagasaki University, Nagasaki, Japan; ^3^ Shionogi Global Infectious Diseases Division, Institute of Tropical Medicine, Nagasaki University, Nagasaki, Japan

**Keywords:** γδ T cells, cDC1, Th1 cell response, spleen, malaria, blood-stage infection, *Plasmodium*

## Abstract

γδ T cells facilitate the CD4^+^ T helper 1 (Th1) cell response against *Plasmodium* infection by activating conventional dendritic cells (cDCs), although the underlying mechanism remains elusive. Our study revealed that γδ T cells promote the complete maturation and production of interleukin-12 and CXCR3-ligands specifically in type 1 cDCs (cDC1), with minimal impact on cDC2 and monocyte derived DCs (Mo-DCs). During the initial infection phase, γδ T cell activation and temporal accumulation in the splenic white pulp, alongside cDC1, occur via CCR7-signaling. Furthermore, cDC1/γδ T cell interactions in the white pulp are amplified through CXCR3 signaling in γδ T cells, optimizing Th1 cell priming by cDC1. We also demonstrated how transitional Th1 cells arise in the white pulp before establishing their presence in the red pulp as fully differentiated Th1 cells. Additionally, we elucidate the reciprocal activation between γδ T cells and cDC1s. These findings suggest that Th1 cell priming is orchestrated by this reciprocal activation in the splenic white pulp during the early phase of blood-stage *Plasmodium* infection.

## Introduction

Malaria, caused by *Plasmodium* parasite infection, remains a major cause of mortality worldwide, with 608,000 deaths reported in 2022 (1). The immune response against malaria involves various components of the immune system. CD4^+^ T cells are the major drivers of protection against blood-stage infection in mouse model of malaria (2, 3). During this phase, CD4^+^ T cells differentiate into T helper 1 (Th1) cells or T follicular helper (Tfh) cells, which are influenced by signals from antigen-presenting cells (APCs) such as conventional dendritic cells (cDCs) (4–6). Th1 cells are a major source of interferon-γ (IFN-γ) during blood-stage infection, enhancing the parasite-killing function of macrophages (7). By contrast, Tfh cells facilitate B cell affinity maturation and humoral immunity (8).

cDCs serve as a link between innate and adaptive immune responses. In mice, cDCs comprise two subsets with distinct functions: CD8α^+^ XCR1^+^ type 1 cDC (cDC1) and CD11b^+^ type 2 cDC (cDC2) (9, 10). cDC1 is important for promoting *Plasmodium*-specific Th1 and Tfh cells (11, 12). Although both cDC1 and cDC2 isolated from *Plasmodium*-infected mouse spleens can promote IFN-γ production from CD4^+^ T cells *in vitro*, only cDC2 showed the unique ability to promote the production of IL-4 and IL-10 (13).

γδ T cells are major players in the immune response to *Plasmodium* infection, with γδ T cell-deficient mice often exhibiting impaired parasite clearance (2, 14–16). γδ T cells expand in the later phase of blood-stage *Plasmodium* infection (2, 15–17) and suppress parasitemia in a macrophage colony-stimulating factor (M-CSF)-dependent manner in the late phase of blood-stage *Plasmodium chabaudi* infection (2, 15). On the other hand, γδ T cells play a crucial role in the early phase of blood-stage attenuated-*Plasmodium berghei* infection through CD40L-mediated interaction with cDCs, essential for enhancing IFN-γ production from Th1 cells for parasite control (2, 14, 18). However, the cDC subset responsible for mediating this effect of γδ T cells on Th1 priming remains unknown.

The spleen serves as the central hub for immunity against blood-borne pathogens, including blood-stage *Plasmodium* infection (2, 19). The spatiotemporal organization of immune cells within the spleen is governed by the interactions of various chemokines and their receptors. For example, the chemokine receptor CCR7 is required for the localization of naïve αβ T cells within the T cell zone in the white pulp (20), as well as for guiding antigen-carrying mature cDCs into the white pulp (21). This co-localization of naïve T cells with cDCs is essential for optimal priming (22). The expression of chemokine receptors on activated CD4^+^ T cells can also influence their differentiation by promoting interactions with specific APCs (23). CXCR3 and CXCR5 are involved in Th1 and Tfh differentiation, respectively. CXCR3 supports the interaction of CD4^+^ T cells with CXCL9/CXCL10-producing cDCs (24), while CXCR5 guides Tfh cells to B cell follicles (25–27). *Plasmodium*-specific CD4^+^ T cells undergo a transitional state in which they express both CXCR3 and CXCR5 before differentiating into Th1 or Tfh cells (4). Although the splenic localization of conventional T cells and cDCs and factors dictating their localization are well understood, the localization of γδ T cells remains more ambiguous. Although γδ T cells are primarily found in the splenic red pulp (28, 29), some studies have detected these cells in the splenic white pulp (30, 31). γδ T cells express a wide range of chemokine receptors. CCR7 is expressed in γδ T cells in a similar fashion to αβ T cells (32), although CCR7 appears unnecessary for γδ T cell migration from the skin to the draining lymph nodes (33). CXCR3 is involved in the trafficking of γδ T cells to inflammation sites (34). The splenic localization of γδ T cells under steady-state conditions and following *Plasmodium* infection, and the regulation of this localization by chemokine receptors require further investigations.

In this study, we demonstrated the role of γδ T cells in initiating Th1 commitment during the early stages of malaria. Our findings reveal a reciprocal activation between γδ T cells and cDC1, both of which transiently accumulate in the splenic white pulp during the early phase of infection. The transient relocation of cDC1 to the white pulp is regulated by the upregulation of CCR7. γδ T cells, on the other hand, proliferate in the white pulp and express CCR7, contributing to their transient accumulation. Enhanced interactions between γδ T cells and cDC1 in the white pulp are facilitated via the CXCR3/CXCL9 axis. Notably, cDC1, as the primary IL-12-producing DC subset, is influenced by γδ T cell deficiency. Consequently, γδ T cells create an optimal environment for Th1 priming of *Plasmodium*-specific CD4^+^ T cells, with minimal impact on Tfh priming. Analysis of IFN-γ reporter PbT-II cells revealed the presence of CXCR5^+^IFN-γ^+^ transitional Th1 cells, indicating their origin in the splenic white pulp, distinct from fully differentiated CXCR5^−^ Th1 cells observed in the red pulp. Given the protective role of γδ T cells in *Plasmodium* infection, their capacity to induce Th1 commitment holds promise for advancing strategies in malaria vaccine development.

## Materials and methods

### Mice and parasites

All mice were maintained in the Laboratory Animal Center for Animal Research at the Nagasaki University. C57BL/6 mice were purchased from SLC (Shizuoka, Japan). MHCII restricted-TCR transgenic mice “PbT-II mice” were previously described (11). PbT-II mice were bred with CD45.1^+^ C57BL/6 mice to obtain CD45.1^+^ PbT-II mice. IFN-γ^eYFP^ reporter mice and CD11c^DTR^ mice were purchased from The Jackson Laboratory. Additionally, CD45.1^+^ PbT-II mice were bred with IFN-γ^eYFP^ to obtain CD45.1^+^ PbT-II/IFN-γ^eYFP^ mice. TCRδ KO mice were obtained from RIKEN BioResource Research Center. Age matched mice between 8 and 12 weeks old were used in all experiments. The animal experiments were approved by the Institutional Animal Care and Use Committee of Nagasaki University (Approval #2205171789-7) and were conducted in compliance with the guidelines for Animal Experimentation of Nagasaki University.

*P. chabaudi AS* was kindly provided by Dr. R. Culleton (Ehime University, Ehime, Japan). *P. berghei* ANKA was kindly provided by DR. Masao Yuda (Mie University). Parasites passed through a stock mouse from a frozen stock. And mice were infected intraperitoneally (ip) with 5×10^4^ infected red blood cells (iRBCs). Parasitemia was determined by microscopy using standard thin blood smears stained with a Diff-Quick staining kit (Sysmex, Kobe, Japan).

### Generation of bone marrow chimera mice

Bone marrow was extracted from CD11c^DTR^ donor mice, then 5×10^6^ cells were injected into the tail vein of each 900 Gy irradiated recipient C57BL/6 mice. Experiments were initiated 8-16 weeks after reconstitution.

#### *In vivo* DC depletion

For *in vivo* depletion of dendritic cells (DCs), CD11c^DTR^ bone marrow chimera mice received 100 ng of DT via ip every other day, starting from Day -1.

#### *In vivo* antibody labeling

Mice were injected with 15 μg of FITC anti-mouse CD45.2 Antibody (Biolegend) via the tail vein and sacrificed for analysis 5 minutes later. Spleens were quickly obtained and processed as described later.

### Cell isolation

Spleens were extracted and physically digested in R5 medium (RPMI 1640 medium, 5% fetal bovine serum (FBS), 2 mM glutamine, 1mM sodium pyruvate, 50 μM β-mercaptoethanol, penicillin, streptomycin, and non-essential amino acids 0.1mM). Splenocyte suspension was strained through 70 μm mesh. Gey’s solution was used to lyse RBCs, and splenocytes were finally washed and incubated with Fc block for 15 minutes prior to antibody staining.

Blood samples were collected from the tail vein of living mice for monitoring purposes, or from the inferior vena cava of euthanized mice. Gey’s solution was used to lyse RBCs, and lymphocytes were washed and stained with antibodies.

### PbT-II cells adoptive transfer

PbT-II cells were harvested from spleens of PbT-II mice by using anti-CD4 iMag magnetic beads (BD Biosciences). Host mice received 1×10^6^ PbT-II cells via tail vein injection.

### Flowcytometry

Cell suspensions were stained with the indicated antibodies (see table) in iMag buffer for 30 minutes at 4 °C (for surface markers), except for CCR7 which was incubated at 37 °C. 7AAD was used to identify dead cells.

For intracellular staining of transcription factors, following surface marker staining, cells were fixed and permeabilized using Foxp3 transcription factor staining buffer kit (eBioscience) and stained subsequently according to the kit’s instructions.

For intracellular cytokine staining, cells were cultured in R10 medium (RPMI 1640 medium, 10% FBS, 2 mM glutamine, 1 mM sodium pyruvate, 50 μM β-mercaptoethanol, penicillin, streptomycin, and non-essential amino acids 0.1 mM) for 4 hours, in the presence of Brefeldin A 10 μg/ml. 24-well flat-bottom plates were used. Each well contained 2×10^6^ splenocytes in experiments with no stimulation.

For experiments with PMA/Ionomycin stimulation, 24-well flat-bottom plates were used. Each well contained 2×10^6^ splenocytes. PMA was added at a 25 ng/ml, and ionomycin was added at a 1 μg/ml.

For experiments with PbT-II peptide stimulation, DCs were sorted from uninfected mice using AutoMACS (Miltenyi Biotec) utilizing CD11c microbeads and cultured in a 96 round bottom well plate (10^4^ cells per plate) in R10 medium with PbT-II peptide 1μM (DNQKDIYYITGESINAVS) (Sigma-Aldrich) (35) for 1 hour. Simultaneously, CD4^+^ T cells were isolated from splenocyte suspension using anti-CD4 iMag magnetic beads (BD Biosciences). 2×10^5^ CD4^+^ T cells were then added to the pulsed DC cell suspension in each well, and Brefeldin A was added at a 3 μg/ml. The cells were then cultured for 4 hours.

Cells were stained for surface markers, then were washed, fixed and permeabilized using BD Cytofix/Cytoperm™ PlusFixation/Permeabilization Kit (BD Biosciences), and stained subsequently according to instructions.

Stained cell suspensions were analyzed using LSRFortessa X-20 cell analyzer and FlowJo software (version 10.8.2).

### Immunohistochemistry and confocal microscopy

Spleens were extracted and embedded in OCT medium (Sakura Finetek, Tokyo, Japan), snap-frozen using methanol and dry ice, and stored at -80 °C. 7 μm thick spleen sections were then obtained using a cryomicrotome (Leica, Germany). Sections were dried for an hour, fixed in -20 °C acetone for 15 minutes, washed in PBS, and blocked using Blocking One Histo (Nacalai, Kyoto, Japan) for 1 hour in room temperature. Subsequently, sections were stained using fluorescent antibodies in blocking buffer overnight at -4 °C. The next day, sections were washed with PBS and stained with DAPI for 5 minutes in room temperature, followed by washing. Sections were finally mounted in DAKO fluorescent mounting medium (Agilent Technologies, Santa Clara, CA, USA).

For immunohistochemistry with splenic sections of IFN-γ^eYFP^ reporter mice, paraformaldehyde (PFA) perfusion was performed as described by previous report (36). Mice were put in deep anesthesia using a lethal dose of a mixture of Dorbene vet, Midazolam, and Vetorphale. Their circulatory systems were then perfused with ice cold PBS, followed by perfusion of 4% PFA. Spleens were then extracted and submerged in 4% PFA for 2 hours followed by dehydration using 15% and 30% sucrose solutions respectively overnight. Spleens were then dried, embedded in OCT medium, frozen, stored at -80 °C. Splenic sections were obtained as stated above. The sections were rehydrated in PBS containing 0.3% Tween 80 for an hour, blocked, and subsequently stained as stated above.

Images of stained sections were obtained using LSM 800 confocal microscope system (Carl Zeiss, Jena, Germany) and merged using the Fiji software (National Institutes of Health, Bethesda, MD, USA). Colocalization Object Counter plugin (37) was used to count cells in images.

### Statistical analysis

GraphPad Prism (Prism version 8, La Jolla, CA, USA) was used to perform statistical analysis. Two-tailed unpaired Student’s t-tests were used to compare two groups. For analyses involving 3 groups, one-way ANOVA was used, and the differences between individual groups was determined by Tukey’s test.

## Results

### γδ T cells promote Th1 differentiation in *Plasmodium*-specific CD4^+^ T cells during the acute phase of malaria

**Figure 1:**
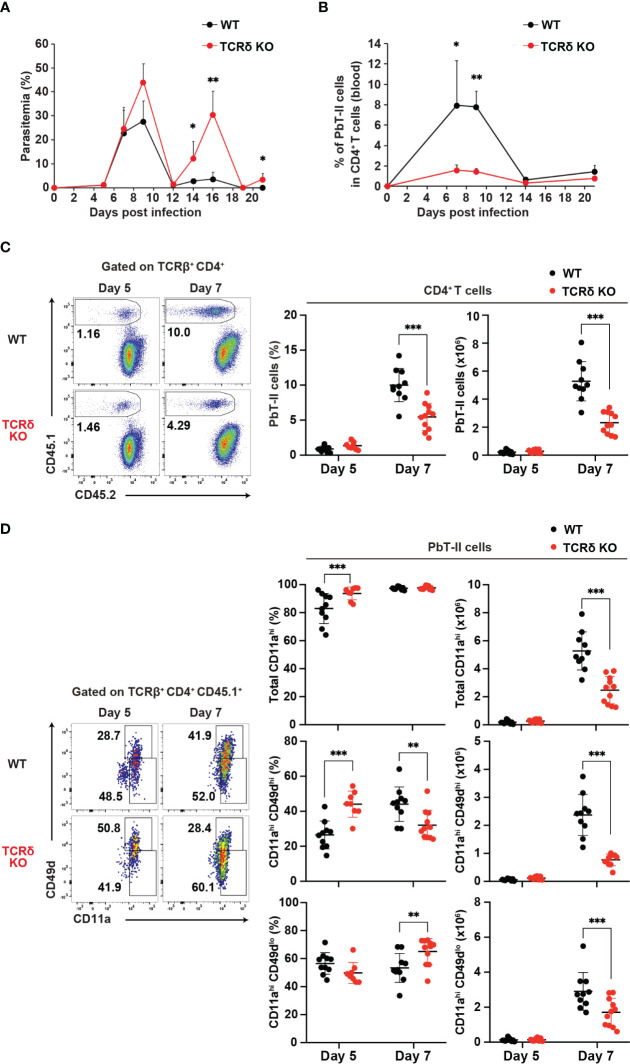
γδ T cells promote the activation of *Plasmodium*-specific CD4^+^ T cells during the acute phase of malaria. PbT-II cells were transferred into WT and TCRδ KO mice 1 day prior to infection with *P. chabaudi*. **(A, B)** Parasitemia **(A)** and PbT-II cells **(B)** were monitored in peripheral blood. **(C)** Representative flow plot (left), proportion (center), and number (right) of PbT-II cells (CD45.1^+^) in the spleen. **(D)** Representative flow plot (left), proportions (center), and numbers (right) of total CD11a^hi^, CD11a^hi^CD49d^hi^, and CD11a^hi^CD49d^lo^ cells in splenic PbT-II cells. ∗p < 0.05; ∗∗p < 0.01; ∗∗∗p < 0.001; Student’s t-test. Each symbol represents an individual mouse, while error bars indicate SD. Data in **(A, B)** are representative of two experiments, with each group comprising 3–4 mice. Data in **(C, D)** were pooled from 8–11 mice per group from seven experiments. (See also **Supplementary Figure 1**).

To evaluate the response of *Plasmodium*-specific CD4^+^ T cells during *Plasmodium* infection, we utilized the *Plasmodium*-specific T cell receptor (TCR)-transgenic (PbT-II) mouse line (11). CD4^+^ PbT-II cells were transferred into wild-type (WT) and TCRδ knockout (KO) mice, and we monitored parasitemia levels and peripheral blood PbT-II cells after infection with *P. chabaudi* or *P. berghei* ANKA (**Supplementary Figures 1A, B**). During the first week of infection with *P. chabaudi* or *P. berghei* ANKA, there was no significant difference in parasitemia levels between the mouse groups. However, TCRδ KO mice exhibited delayed parasite clearance at later time points of *P. chabaudi* infection compared to WT mice, consistent with previous observations (**Figure 1A**) (15, 38, 39). In the case of *P. berghei* ANKA infection, a model for cerebral malaria, TCRδ KO mice showed approximately 30% reduction of cerebral malaria development despite comparable parasitemia levels to WT mice (40, 41) (**Supplementary Figures 1C, D**). The results of *P. berghei* ANKA infection suggest the influence of γδ T cells on the immune response during the acute phase of infection. The proportions and numbers of PbT-II cells in the blood and spleen increased in both groups on day 7 post-infection (pi) with *P. chabaudi*, but were significantly lower in TCRδ KO mice (**Figures 1B, C**). This impaired expansion of PbT-II cells in TCRδ KO mice was also evident following *P. berghei* ANKA infection, both in peripheral blood and spleen (**Supplementary Figures 1E, F**). Despite these results, most splenic PbT-II cells from both mouse groups exhibited high expression of the activation marker CD11a during the acute phase of *P. chabaudi* infection (**Figure 1D**). However, the proportions and numbers of CD11a^hi^CD49d^hi^, indicative of a Th1-cell phenotype (42–44), were significantly lower in splenic PbT-II cells from TCRδ KO mice on day 7 pi compared to WT mice. Conversely, CD11a^hi^CD49d^lo^, representing a Tfh-cell phenotype (42), showed higher proportions but lower numbers in splenic PbT-II cells from TCRδ KO mice on day 7 pi (**Figure 1D**). Similar activation markers were assessed on day 5 pi, revealing a significantly higher proportion of total CD11a^hi^ and CD11a^hi^CD49d^hi^ populations in TCRδ KO mice (**Figure 1D**). This suggests that the initial activation of PbT-II cells was not impaired in the absence of γδ T cells.

**Figure 2:**
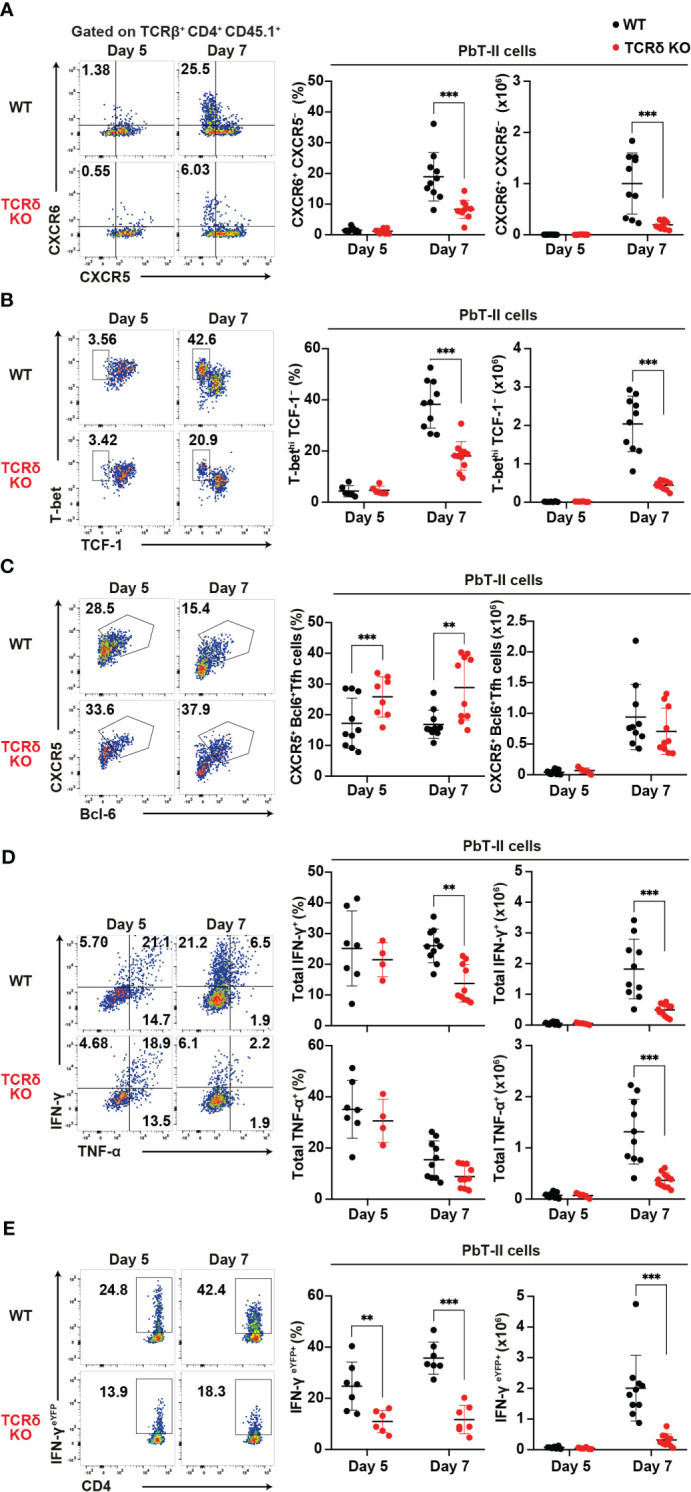
γδ T cells enhance the Th1 immune response in *Plasmodium*-specific CD4^+^ T cells during the acute phase of malaria. PbT-II cells or IFN-γ^eYFP^ PbT-II cells were transferred into WT and TCRδ KO mice 1 day prior to infection with *P. chabaudi*. **(A)** Representative flow plot (left), proportion (center), and number (right) of CXCR6^+^ PbT-II cells. Data were pooled from 8–11 mice per group from seven experiments. **(B)** Representative flow plot (left), proportion (center), and number (right) of T-bet^hi^ TCF1^–^ in PbT-II cells. Data were pooled from 6–11 mice per group from six experiments. **(C)** Representative flow plot (left), proportion (center), and number (right) of CXCR5^+^ BCL6^+^ PbT-II cells. Data were pooled from 8–10 mice per group from seven experiments. **(D)** Representative flow plot (left), proportion (center), and number (right) of total IFN-γ^+^ and total TNFα^+^ in splenic PbT-II cells following cell culture with PbT-II peptide pulsed DCs. Data were pooled from 4–10 mice per group from six experiments. **(E)** Representative flow plot (left), proportion (center), and number (right) of eYFP^+^ in IFN-γ^eYFP^ PbT-II cells. Data were pooled from 6–7 mice per group from four experiments. ∗∗p < 0.01; ∗∗∗p < 0.001; Student’s t-test. Each symbol represents an individual mouse, while error bars indicate SD.

Following blood-stage *Plasmodium* infection, CD4^+^ T cells primarily differentiate into Th1 or Tfh cells (4). To confirm aberrant T cell differentiation in TCRδ KO mice during the acute phase of infection, we conducted a phenotypic analysis of PbT-II cells. On day 7 pi, splenic PbT-II cells in TCRδ KO mice exhibited significantly lower proportions of CXCR6^+^CXCR5^–^ and Tbet^hi^TCF1^–^, which are canonical Th1-cell markers (45–47), compared to those in WT mice (**Figures 2A, B**). However, these differences were not observed on day 5 pi, when the proportion and number of splenic PbT-II cells were comparable between the groups (**Figure 1**). In contrast, splenic PbT-II cells in TCRδ KO mice demonstrated significantly higher proportions of CXCR5^+^Bcl-6^+^, canonical Tfh cell markers (48), although the numbers of splenic Tfh cells were comparable between the two groups (**Figure 2C**). Furthermore, to assess the production of Th1-related cytokines in PbT-II cells, we employed an *in vitro* culture system with peptide-pulsed DCs and an *ex vivo* system with IFN-γ^eYFP^ reporter PbT-II cells. The production of IFN-γ in cultured splenic PbT-II cells from TCRδ KO mice was significantly lower on day 7 pi compared to that in WT mice, with a similar trend observed in TNF-α expression (**Figure 2D**). Additionally, IFN-γ^eYFP^ reporter splenic PbT-II cells in TCRδ KO mice exhibited lower expression of IFN-γ than those in WT mice on both day 5 and day 7 pi (**Figure 2E**). The impaired IFN-γ production of PbT-II cells in TCRδ KO mice on day 5 pi *ex vivo*, but not *in vitro* when stimulated by naïve DCs, indicates a possible deficient stimulation for PbT-II cells by cDCs in TCRδ KO mice at day 5 pi (**Figures 2D, E**). The IFN-γ^high^ population of splenic PbT-II cells predominantly exhibited a CXCR6^+^CXCR5^–^ Th1 phenotype in both WT and TCRδ KO mice on day 7 pi (**Supplementary Figure 1G**). Collectively, these results suggest that γδ T cells play a critical role in promoting Th1 cell differentiation of *Plasmodium*-specific CD4^+^ T cells in the acute phase of infection.

### γδ T cells affect cDC1 function during the initial phase of malaria

**Figure 3:**
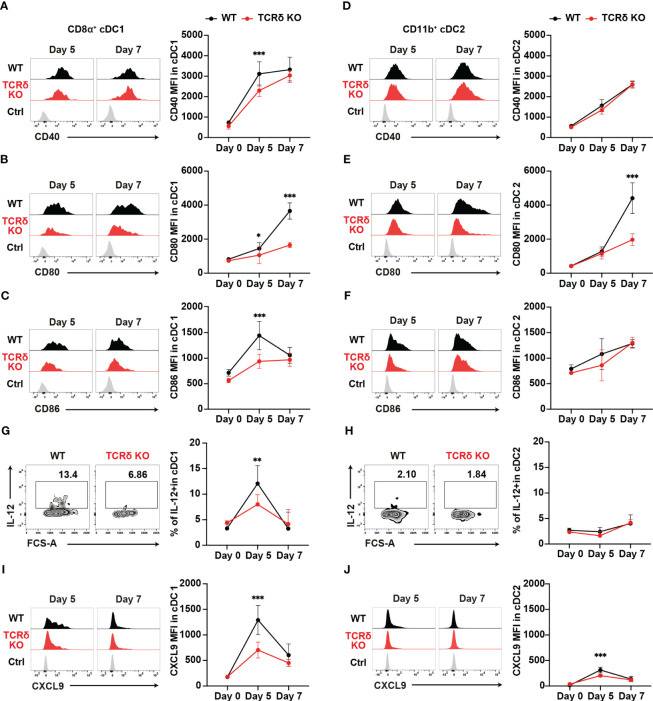
γδ T cells enhance the functional maturation of cDC1 during the initial phase of malaria. WT and TCRδ KO mice were infected with *P. chabaudi*. **(A–C)** Representative histogram (left) and average MFI (right) of CD40 **(A)**, CD80 **(B)**, and CD86 **(C)** in splenic CD8α^+^ cDC1. The gating strategy is shown in **Supplementary Figures 3A–C**. **(D–F)** Representative histogram (left) and average MFI (right) of CD40 **(D)**, CD80 **(E)**, and CD86 **(F)** in splenic CD11b^+^ cDC2 cells. The gating strategy is in **Supplementary Figures 3A–C**. **(G, H)** Representative histograms (left) and proportions of IL-12^+^ cells in cDC1 **(G)** and cDC2 **(H)** cells. **(A–H)** Data were pooled from 3 mice per group (day 0) from one experiment, 7–13 mice per group (day 5) from four experiments, and 6 mice per group (day 7) from two experiments. **(I, J)** Representative histograms (left) and proportions of CXCL9^+^ in cDC1 **(I)** and cDC2 **(J)** respectively. Data were pooled from 3 mice per group (day 0) from one experiment and 8 mice per group (day 5) from two experiments and 5 mice per group (day 7) from one experiment. ∗p < 0.05; ∗∗p < 0.01; ∗∗∗p < 0.001; Student’s t-test. Symbols represent the mean values, while error bars indicate the SD. **(A–F, I, J)** Ctrl histograms represent the isotype controls. See also **Supplementary Figure 2**.

cDCs are known for their pivotal role in priming naïve T cells and shaping T helper subset commitment (11, 49, 50). To unravel the mechanisms behind the impaired Th1 differentiation of *Plasmodium*-specific CD4^+^ T cells in TCRδ KO mice, we conducted an analysis of cDC subsets (**Supplementary Figure 2A**). The initial activation of PbT-II cells was observed from day 5 pi (**Figures 2C, D**), indicating the commencement of CD4^+^ T cell priming by cDCs in the spleen. The numbers of cDCs and the composition of cDC subsets in the spleen were similar between the mouse groups from steady-state condition until day 7 pi (**Supplementary Figures 2B, C**). However, on day 5 pi, splenic cDC1 displayed reduced expression levels of maturation markers such as CD40, CD80, and CD86 in TCRδ KO mice compared to those in WT mice (**Figures 3A–C**). In contrast, the expression of maturation markers in splenic cDC2 from TCRδ KO mice was comparable to that from WT mice on day 5 pi, with differences observed only in lower CD80 expression levels in TCRδ KO mice on day 7 pi (**Figures 3D–F**).

Considering the importance of interleukin-12 (IL-12) in promoting Th1 differentiation (51, 52), we analyzed the production of IL-12 in both splenic cDC subsets. cDC1 showed a transient increase in IL-12 levels on day 5 pi in both mouse groups. However, TCRδ KO mice had significantly lower IL-12-producing cDC1s on day 5 pi (**Figure 3G**). By contrast, cDC2 did not increase IL-12 production on day 5 pi in either group compared with the steady-state levels (**Figure 3H**). Finally, the optimal crosstalk between CD4^+^ T cells and cDCs for priming relies on chemokine/chemokine-receptor interactions. Among Th1 cells, the most prominent chemokine receptor is CXCR3, which facilitates their interactions with cDCs through the production of its ligands, CXCL9 and CXCL10 (24). Furthermore, CXCL9 and CXCL10 signaling through CXCR3 promotes Th1 polarization by inducing the phosphorylation of STAT1, STAT4, and STAT5 (53). Therefore, we analyzed the expression levels of CXCL9 in myeloid cells. CXCL9 expression peaked on day 5 pi in both splenic cDC subsets, with cDC1 showing higher expression levels than cDC2. However, TCRδ KO mice displayed significantly lower CXCL9 levels in both cDC1 and cDC2 at this time point. Subsequently, on day 7 pi, CXCL9 expression decreased in both cDC1 and cDC2, with no significant differences observed between WT and TCRδ KO mice (**Figures 3I, J**). Another subset of CXCL9-producing myeloid cells was CD11c^int^MHCII^+^CD11b^hi^ monocyte-derived DC (Mo-DC). Mo-DCs, which are derived from inflammatory monocytes, are known to promote Th1 differentiation (54). Previous studies have shown that inflammatory monocytes are the major producers of CXCL9 in blood-stage *Plasmodium* infection (4). Analysis of splenic Mo-DCs revealed an increase in their numbers on day 5 pi, with no significant difference observed between WT and TCRδ KO mice (**Supplementary Figure 2D**). However, splenic Mo-DCs in TCRδ KO mice displayed reduced expression levels of the maturation marker CD86, while CD40 and CD80 levels were similar to those in WT mice on day 5 pi (**Supplementary Figures 2E–G**). CXCL9 expression in Mo-DCs peaked on day 5 pi and subsequently decreased on day 7 pi. TCRδ KO mice showed significantly lower CXCL9 levels in Mo-DCs on day 5 pi peak, mirroring the same dynamic observed in cDCs. However, CXCL9 expression in Mo-DCs did not reach the levels observed in cDC1 (**Supplementary Figure 2E**). Collectively, these results suggest that γδ T cells play a critical role in the maturation and IL-12 production of cDC1, but not cDC2. Moreover, they promote CXCL9 production from both cDCs and Mo-DCs.

### γδ T cells are activated in the acute phase of malaria

**Figure 4:**
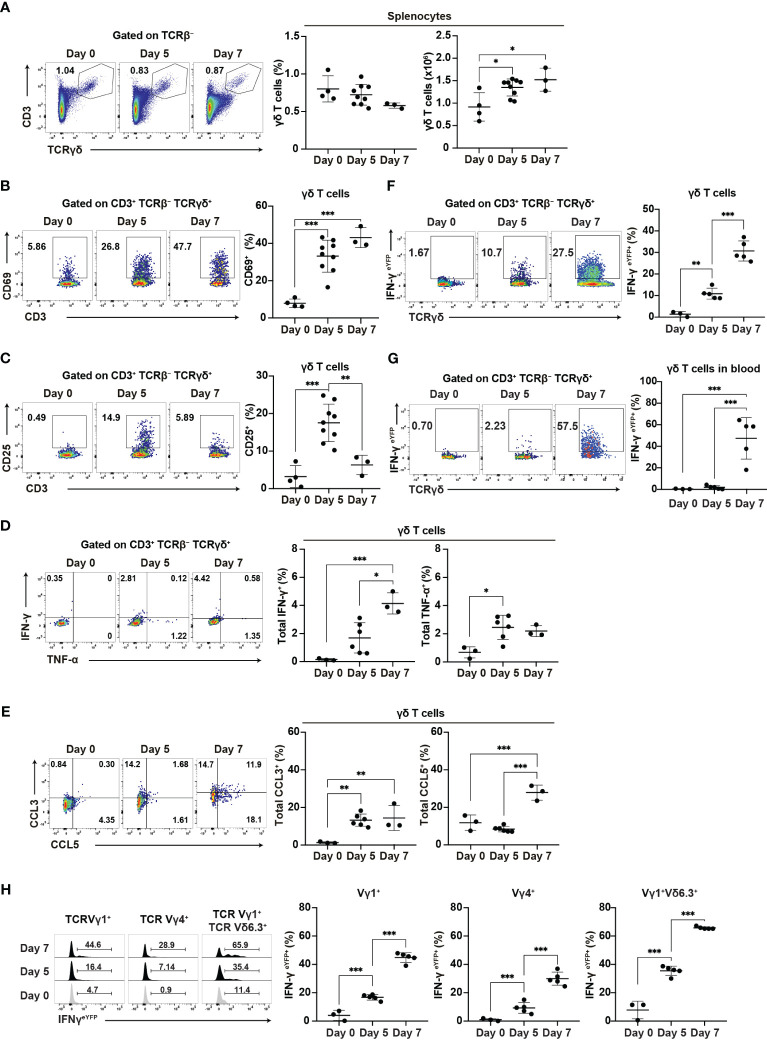
γδ T cells are activated during the initial phase of malaria. WT mice or IFN-γ^eYFP^ mice were infected with *P. chabaudi*. **(A)** Representative flow plots (left), proportion (center), and number (right) of T cells in the spleen. **(B, C)** Representative flow plots (left) and proportion (right) of CD69^+^ **(B)** and CD25^+^ **(C)** in splenic γδ T cells. **(A–C)** Data were pooled from 3–9 mice per group from four experiments. **(D)** Representative flow plots (left) and proportion of total IFN-γ^+^ (center) and total TNF-α^+^ (right) in splenic γδ T cells following cell culture with no stimulation. **(E)** Representative flow plots (left) and proportion of total CCL3^+^ (center) and total CCL5^+^ (right) in splenic γδ T cells following cell culture with no stimulation. **(D, E)** Data were pooled from 3–6 mice per group from three experiments. **(F, G)** Representative flow plots (left) and proportion of eYFP^+^ (right) in γδ T cells in the spleen **(F)** and blood **(G)** from IFN-γ^eYFP^ mice. **(H)** Representative flow plots (left) and proportion of eYFP^+^ (right) within splenic γδ T cell subsets from IFN-γ^eYFP^ mice. **(F–H)** Data were pooled from 3–5 mice per group from three experiments. ∗q<0.05; ∗∗q<0.01; ∗∗∗q<0.001; One-way ANOVA and Tukey’s multiple comparison test. Each symbol represents an individual mouse, while error bars indicate the SD. See also **Supplementary Figure 3**.

Our findings suggest the important role of γδ T cells in the early phase of *Plasmodium* infection. Therefore, we conducted a phenotypic analysis of γδ T cells during this early phase. The proportion of γδ T cells did not increase in the spleen up to day 7 pi, while γδ T cell numbers increased from day 5 pi (**Figure 4A**). Canonical T cell activation markers showed various expression patterns in splenic γδ T cells. CD69 and CD11a expression gradually increased and persisted during the acute phase, from day 5 pi (**Figure 4B**, **Supplementary Figure 3A**), while CD49d expression remained unchanged (**Supplementary Figure 3A**). By contrast, CD25 expression transiently increased on day 5 pi (**Figure 4C**), while PD1 expression increased on day 7 pi (**Supplementary Figure 3B**).

The ability of γδ T cells for cytokine production is crucial for their function. Additionally, γδ T cells are a major source of chemokines such as CCL3 and CCL5 in the later phase of blood-stage *P. chabaudi* infection and those chemokines are thought to facilitate interaction with myeloid cells (15). Therefore, we examined the expression of cytokines and chemokines in splenic γδ T cells. Unstimulated γδ T cells showed an increase in IFN-γ expression from day 5 pi, although this increase only reached the significance level on day 7 pi. γδ T cells significantly increased TNF-α and CCL3 expression from day 5 pi. CCL5 expression was also increased on day 7 (**Figures 4D, E**). Under phorbol 12-myristate 13-acetate (PMA)/ionomycin stimulation, γδ T cells exhibited further enhancement of cytokine and chemokine production. However, PMA/ionomycin stimulation may have masked the onset of expression (**Supplementary Figures 3C, D**). Furthermore, using IFN-γ^eYFP^ mice, we confirmed that the IFN-γ production of splenic γδ T cells started on day 5 pi, with a substantial increase observed on day 7 pi (**Figure 4F**). In contrast to splenic γδ T cells, peripheral blood γδ T cells demonstrated a delayed response, with the first significant increase in IFN-γ production occurring on day 7 pi (**Figure 4G**). These results suggest that the activation of γδ T cells started in the spleen from the initial phase of blood-stage *Plasmodium* infection.

Finally, we examined the major γδ T cell subsets in the spleen (Vγ1^+^ and Vγ4^+^). Among these, the Vγ1^+^Vδ6.3^+^ subset has been shown to expand and play a major role in the later phase of *P. chabaudi* infection (15). However, we did not observe a specific increase in the proportion of Vγ1^+^Vδ6.3^+^ subset in the spleen up to day 7 pi (**Supplementary Figure 3E**). Each γδ T cell subset exhibited an increased expression of CD69 and IFN-γ (**Supplementary Figures 3F, 4H**). However, the Vγ1^+^Vδ6.3^+^ subset showed the highest levels of CD69 and IFN-γ. These results suggest that despite the global activation of different γδ T cell subsets, the Vγ1^+^Vδ6.3^+^ subset might coordinate the development of Th1 immune response.

### γδ T cells influence the splenic localization of *Plasmodium*-specific CD4^+^ T cells and cDCs during the acute phase of malaria

**Figure 5:**
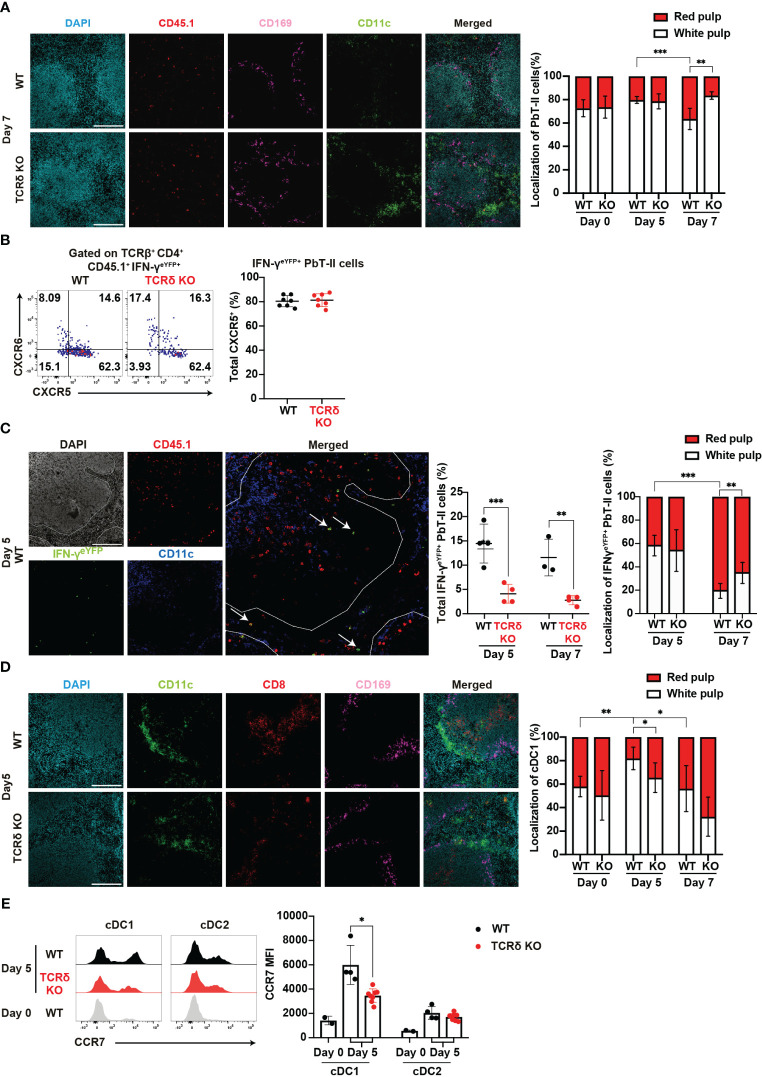
γδ T cells influence the splenic localization of key cell subsets during the acute phase of malaria. **(A–C)** PbT-II cells or IFN-γ^eYFP^ PbT-II cells were transferred into WT and TCRδ KO mice 1 day prior to infection with *P. chabaudi*. **(A)** Representative images of fluorescence-labeled-section from the spleen on day 7 pi (left) and summary of the localization of PbT-II cells (right) in the spleen from WT and TCRδ KO mice. Scale bars measure 200 μm. Data were pooled from 3–5 mice per group. **(B)** Representative flow plots (left) and proportion (right) of CXCR5^+^ in IFN-γ^eYFP+^ cells in the spleen on day 5 pi. **(C)** Representative image of WT mice on day 5 pi (left), proportion of total IFN-γ^eYFP+^ PbT-II cells (center), and localization of IFN-γ^eYFP+^ PbT-II cells (right) in the spleen from WT and TCRδ KO mice on day 5 and day 7 pi. The white line outlines the white pulp borders, and arrowheads point to the examples of IFN-γ^eYFP+^ PbT-II cells. Scale bars measure 200 μm. Data pooled from 3-4 mice per group. **(D, E)** WT and TCRδ KO mice were infected with *P. chabaudi*. **(D)** Representative images (left) of fluorescence-labeled section from the spleen on day 5 pi, and summary of localization of cDC1 (right) in WT and TCRδ KO mice. Scale bars measure 200 μm. Data pooled from 3–6 mice per group. **(E)** Representative images (left) and MFI (right) of CCR7 expression in cDC1 and cDC2. Data pooled from 2 WT mice on day 0, and 4–6 mice per group on day 5 pi, from two experiments. ∗p<0.05; ∗∗p<0.01; ∗∗∗p<0.001 **(B, C, E)** Student’s t-test was used. **(A, D)** One-way ANOVA and Tukey’s multiple comparison test were performed. ∗q<0.05; ∗∗q<0.01; ∗∗∗q<0.001; each symbol represents an individual mouse; error bars indicate the SD. See also **Supplementary Figure 4**.

The distribution of immune cells within the spleen is intricately linked to their function. Thus, the analysis of this distribution may provide insights into the immune landscape during the early phase of infection. Following *Plasmodium* infection, Tfh-type PbT-II cells are localized in the white pulp, while Th1-type PbT-II cells are localized in the red pulp³². Considering the impaired Th1 differentiation in TCRδ KO mice, we expected a subsequent effect on the splenic distribution of PbT-II cells. Analysis of spleen sections from WT and TCRδ KO mice showed an increase in the proportion of red pulp-localized PbT-II cells in WT mice but not in TCRδ KO mice on day 7 pi (**Figure 5A**). These results were consistent with the observed Th1 differentiation in both mouse models (**Figures 2A, B**). Although the Th1 differentiation of PbT-II cells occurred on day 7 pi (**Figures 2A, B**), IFN-γ^eYFP^-expressing PbT-II cells were readily detectable in the spleen on day 5 pi (**Figure 2E**). These cells primarily exhibited the CXCR5^+^CXCR6^−^ phenotype (**Figure 5B**), suggesting that they had not yet fully differentiated into Th1 cells. The IFN-γ^eYFP^-expressing cells detected on day 5 pi were referred to as “transitional Tfh-Th1 (trans-Th1)” cells (55). To clarify splenic localization of the trans-Th1 cells, we analyzed spleen sections obtained from WT and TCRδ KO mice that received IFN-γ^eYFP^ reporter PbT-II cells. More than half of the trans-Th1 cells were localized in the white pulp in both WT and TCRδ KO mice on day 5 pi (**Figure 5C**). On the other hand, IFN-γ^eYFP+^ fully differentiated Th1 cells showed preferential localization in the splenic red pulp as previously reported (42), although this was less pronounced in TCRδ KO mice compared to WT mice (**Figure 5C**). These findings suggest that Th1 differentiation was predominantly initiated in the white pulp and that the localization shift of Th1 cells to the red pulp occurred after full differentiation.

Another cell subset profoundly affected by the absence of γδ T cells was cDC1. These cells are normally distributed in the red and white pulp under steady-state conditions. However, following inflammation, cDC1s migrate from the red pulp to the white pulp, placing cDC1 in close proximity to naïve T cells for T cell priming (56, 57). Analysis of cDC1 localization revealed a temporary increase in the proportion of the white pulp-localized cDC1 in WT mice but not in TCRδ KO mice on day 5 pi (**Figure 5D**). This result indicates that γδ T cells are important for the accumulation of cDC1 in the splenic white pulp in the initial phase of infection. The upregulation of CCR7, a hallmark of DC maturation, facilitates their relocation to T cell zones (58, 59). Considering the disrupted cDC1 splenic localization in TCRδ KO mice, we investigated the involvement of CCR7. Indeed, CCR7 expression in cDCs increased on day 5 pi compared with that in the steady state. However, cDC1 in TCRδ KO mice exhibited significantly lower CCR7 expression compared with WT mice (**Figure 5E**). These differences were not observed in cDC2s.

### γδ T cells increased CXCR3-dependent contact with cDC1 in the white pulp during the initial phase of malaria

**Figure 6:**
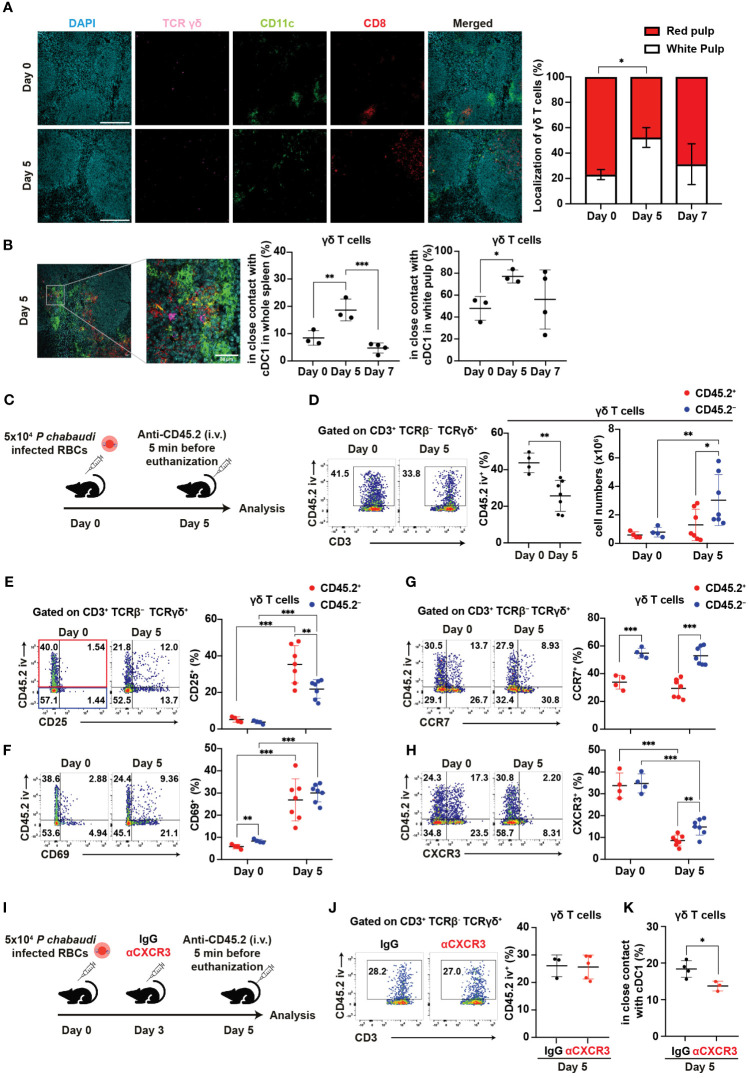
γδ T cells exhibit CXCR3-dependent interaction with cDC1 in the white pulp during the initial phase of malaria. **(A, B)** C57BL/6 WT mice were infected with blood-stage *P. chabaudi*. **(A)** Representative images (left) of fluorescence-labeled section from the spleen on days 0 and 5 pi, and summary of localization of γδ T cells (right) in the spleen. Scale bars measure 200 μm. Data pooled from 3–4 mice per group. **(B)** Representative images (left) of fluorescence-labeled section from the spleen on day 5 pi, proportions (center) of γδ T cells (purple) in close contact with cDC1 (yellow) from the spleen, and percentage of these interactions located in the white pulp (right). Scale bar measures 30 μm. Data pooled from 3–4 mice per group. **(C)** Experiment design for *in vivo* labeling with CD45.2(iv) antibodies. **(D)** Representative flow plots (left), proportion (center) of CD45.2(iv)^+^, and total numbers (right) of CD45.2(iv)^+/–^ splenic γδ T cells. **(E–H)** Representative flow plots (left) and proportions (center) of CD25^+^, CD69^+^, CCR7^+^, and CXCR3^+^ cells in splenic γδ T cells from *in vivo* CD45.2-labeled WT mice. Red dots represent the proportion of the aforementioned cells in CD45.2(iv)^+^ γδ T cells, while blue dots represent their proportion in CD45.2(iv)^–^ γδ T cells. **(C–H)** Pooled data from 3–7 mice per group from three experiments. **(I)** Experiment design for CXCR3 blockade in addition to *in vivo* labeling with CD45.2(iv) antibodies. **(J)** Representative flow plots (left) and summary (right) of the proportions of CD45.2(iv)+ cells in splenic γδ T cells from IgG- and αCXCR3-treated mice on day 5 pi. Data were pooled from 3–5 mice per group from two experiments. **(K)** Proportions of γδ T cells in close contact with cDC1 in the spleen on day 5 pi in mice that received IgG or αCXCR3 treatment. Data were pooled from 3–4 mice per group. **(A, B)** One-way ANOVA and Tukey’s multiple comparison test were performed. ∗q<0.05; ∗∗q<0.01; ∗∗∗q<0.001; each symbol represents an individual mouse; error bars indicate the SD. Student’s t-test was used in the figures **(D–K)**. p<0.05; ∗∗p<0.01; ∗∗∗p<0.001.

Splenic γδ T cells typically reside in the red pulp of the spleen under steady-state conditions (28–30). Indeed, γδ T cells were mainly localized in the red pulp under such conditions, with only a minor population residing in the white pulp. However, the proportion of γδ T cells in the white pulp transiently increased on day 5 pi and subsequently returned to near steady-state levels on day 7 pi (**Figure 6A**). This shift in distribution toward the white pulp resembled cDC1 migration patterns. Hence, we hypothesized an increased cell-cell interaction between γδ T cells and cDC1 on day 5 pi. The proportion of γδ T cells in close contact with cDC1 significantly increased on day 5 pi compared with that at steady-state levels (**Figure 6B**), with interactions predominantly observed in the white pulp.

Next, we investigated the factors behind the change in γδ T cell distribution from steady-state levels to day 5 pi. To analyze the distribution of splenic γδ T cells, we utilized *in vivo* labeling with anti-CD45.2 antibody (**Figure 6C**). *In vivo* anti-CD45.2 labeling identifies immune cells that are accessible to the circulation, including red pulp-localized cells. Consistent with spleen immunohistology data, the proportion of CD45.2(iv)^+^ red pulp-localized γδ T cells significantly decreased on day 5 pi (**Figures 6A, D**). Conversely, the numbers of CD45.2(iv)^–^ white pulp-localized γδ T cells but not CD45.2(iv)^+^ red pulp-localized γδ T cells significantly increased on day 5 pi (**Figure 6D**). To assess the activation status and localization of splenic γδ T cells, we analyzed the expression of activation markers with *in vivo* anti-CD45.2 labeling. The proportions of CD25^+^ and CD69^+^ in both CD45.2(iv)^+^ and CD45.2(iv)^–^ γδ T cells strongly increased on day 5 pi (**Figures 6E, F**). These results suggest that γδ T cells become activated in the white pulp and red pulp, with a possibly more rapid proliferation in the white pulp than in the red pulp during the initial phase of infection.

Next, we investigated whether the chemokine receptor expression of γδ T cells is associated with the increase of white pulp-localized γδ T cells in the initial phase of infection. Previous studies have shown the upregulation of CCR7 in activated human γδ T cells (60). Therefore, we examined the expression of CCR7. CCR7 expression in γδ T cells was not upregulated on day 5 pi. However, CD45.2(iv)^–^ white pulp-localized γδ T cells constitutively showed higher CCR7 expression compared with CD45.2(iv)^+^ red pulp-localized γδ T cells (**Figure 6G**). This result suggests that CCR7 may serve as a constitutive chemokine receptor for γδ T cell recruitment into the white pulp. Additionally, we assessed the expression levels of CXCR5, CXCR6, and CXCR3. CXCR5 expression was almost absent in γδ T cells (**Supplementary Figure 4A**). Conversely, CXCR6 expression increased significantly on day 5 pi in both CD45.2(iv)^+^ and CD45.2(iv)^–^ γδ T cells. On day 5 pi, CD45.2(iv)^–^ white pulp-localized γδ T cells exhibited lower expression of CXCR6 compared with CD45.2(iv)^+^ red pulp-localized γδ T cells (**Supplementary Figure 4B**). Therefore, this redistribution of splenic γδ T cells on day 5 pi is not likely driven by CXCR6. The expression of CXCR3 in γδ T cells was significantly decreased on day 5 pi, although higher CXCR3 expression remained in CD45.2(iv)^–^ white pulp-localized γδ T cells compared with that in CD45.2(iv)^+^ red pulp-localized γδ T cells (**Figure 6H**). This decrease in CXCR3 expression may be due to its degradation following ligand binding (61). To assess the association between CXCR3 in γδ T cells and their accumulation in the white pulp, CXCR3 blockade and *in vivo* labeling with CD45.2 antibodies were performed (**Figure 6I**). The proportion of CD45.2(iv)^+^ red pulp-localized γδ T cells in the CXCR3 blockade group was comparable to that in control (**Figure 6J**). However, the CXCR3 blockade group had a reduced proportion of γδ T cells in close contact with cDC1 (**Figure 6K**). These results suggest that CXCR3 on γδ T cells mediates their interaction with cDC1.

### Activation of γδ T cells requires dendritic cells

**Figure 7:**
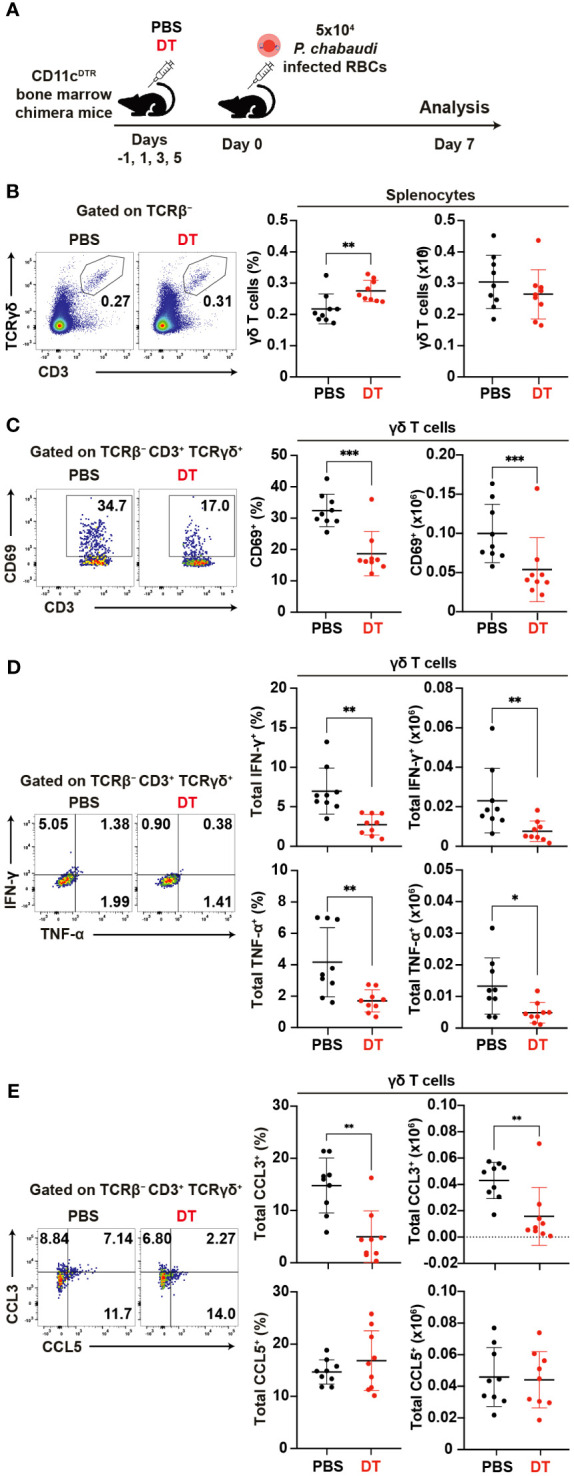
Reciprocal activation of γδ T cells and dendritic cells during the acute phase of malaria. **(A)** Experiment design for cDC depletion. **(B)** Representative flow plots (left), proportion (center), and number (right) of γδ T cells in the spleen on day 7 pi. **(C)** Representative flow plots (left), proportion (center), and number (right) of CD69^+^ in γδ T cells in the spleen on day 7 pi. **(D)** Representative flow plots (left), proportion (center), and number (right) of total IFN-γ^+^ and total TNF-α^+^ in splenic γδ T cells following cell culture with no stimulation on day 7 pi. **(E)** Representative flow plots (left), proportion (center), and number (right) of total CCL3^+^ and CCL5^+^ in splenic γδ T cells following cell culture with no stimulation on day 7 pi. ∗p<0.05; ∗∗p<0.01; ∗∗∗p<0.001; Student’s t-test. Each symbol represents an individual mouse, while error bars indicate the SD. Data were pooled from 9 mice per group from three experiments.

To assess the impact of DC depletion on the activation and function of γδ T cells in the acute phase of *P. chabaudi* infection, we utilized CD11c^DTR^ bone marrow chimeric mice (62)for DC depletion (**Figure 7A**). A substantial reduction in the numbers of splenic cDCs following the administration of diphtheria toxin (DT) was confirmed in the chimeric mice (**Supplementary Figure 5A**). Despite the successful DC depletion, we observed no significant difference in parasitemia between DT-treated group and PBS control group on day 7 pi (**Supplementary Figure 5B**). Although the proportion of splenic γδ T cells increased in the DT group, their numbers on day 7 pi did not differ from those in controls (**Figure 7B**). However, we observed a significant decrease in the expression levels of activation markers in splenic γδ T cells in the DT group (**Figures 7C**, **Supplementary Figures 5C–E**). Additionally, the cytokine and chemokine production from splenic γδ T cells was affected in the absence of DCs. Unstimulated γδ T cells exhibited a significant reduction in IFN-γ and TNF-α expression in the DT group (**Figure 7D**). CCL3 expression was significantly lower in the DT group, while CCL5 expression was comparable between the groups (**Figure 7E**). Results from cell cultures with PMA/ionomycin stimulation corroborated these findings (**Supplementary Figures 5F, G**). Finally, the proportion of Vγ1^+^ γδ T cells increased in the DT group with no changes observed in the remaining subsets (**Supplementary Figure 5H**). CD69 expression was significantly lower in all subsets in the DT group (**Supplementary Figure 5I**). These results suggest that activated DCs play a crucial role in γδ T cell activation during the acute phase of *Plasmodium* infection.

## Discussion

This study elucidates the cellular and molecular landscape governing the initiation of γδ T cell-related protective immune responses during the acute phase of malaria. The accumulation of γδ T cells in the splenic white pulp and their interaction with cDC1 facilitated the optimal Th1 differentiation from *Plasmodium*-specific CD4^+^ T cells. We found that steady-state γδ T cells were predominantly localized in the splenic red pulp, consistent with previous reports (28–30). However, this localization shifted toward the white pulp shortly after the occurrence of infection (on day 5 pi), potentially due to proliferation within this region. Moreover, CCR7 expression was consistently higher in white pulp-localized γδ T cells, suggesting its association with the localization of γδ T cells in the white pulp. On day 5 pi, the splenic γδ T cells showed signs of activation, such as increase of CD69 and CD25 expression, and produced several cytokines and chemokines, such as IFN-γ and CCL3. At this timepoint, increase of IFN-γ production was observed in splenic γδ T cells but not in peripheral blood γδ T cells. These results indicate that γδ T cells are activated in the spleen on day 5 pi, then egress from the spleen to the circulation. The white pulp accumulation of γδ T cells coincided with a similar accumulation of cDC1 in the splenic white pulps of WT mice. Furthermore, these two subsets showed increased close contacts on day 5 pi. CXCR3 expression in γδ T cells significantly decreased after infection, indicating ligand interaction (61). On day 5 pi, cDC1 cells expressed the highest level of CXCL9, which is a CXCR3 ligand (24). This chemokine receptor-ligand interaction proved to be crucial for γδ T cell-cDC1 interactions, as blocking CXCR3 signaling with αCXCR3 antibodies significantly reduced the close contacts between γδ T cells and cDC1 on day 5 pi.

The absence of γδ T cells in TCRδ KO mice resulted in a general disruption in cDC1 function during the early phase of malaria infection. Impairments in costimulatory molecule upregulation and reductions in the levels of cytokines and chemokines necessary to drive Th1 differentiation, such as IL-12 and CXCL9, were observed in cDC1 from TCRδ KO mice. Additionally, the absence of γδ T cells resulted in alterations in the splenic distribution of cDC1. CCR7 upregulation, a hallmark of cDC maturation facilitating migration to the CXCL19/CXCL21-rich T cell zone (58, 59), was impaired in cDC1 from TCRδ KO mice, leading to impaired cDC1 accumulation in the splenic white pulp on day 5 pi. The disruption in cDC1 function in TCRδ KO mice did not extend to cDC2 or Mo-DC function, with most parameters showing comparability between WT and TCRδ KO mice in these subsets. This finding suggests that γδ T cells are not crucial for the function of cDC2 or Mo-DC in malaria infection, and their impact is more pronounced on cDC1 function. This preferential impact of γδ T cells on cDC1 might be due to the bigger role played by cDC1 in priming *Plasmodium*-specific CD4^+^ T cells in response to *Plasmodium* infection compared to other DC subsets (11). A recent study showed that expanded Vγ1^+^Vδ6.3^+^ γδ T cells produce M-CSF in late-phase of *P. chabaudi* infection, resulting in protective role in controlling recrudescent infection (15). M-CSF is known to stimulate myeloid cells. The γδ T cell-derived M-CSF might recruit cDC1 in white pulp of spleen and stimulate cDCs and Mo-DC subsets. Furthermore, various myeloid cell populations, such as CD169^+^ macrophages, and inflammatory monocytes, might be stimulated by the γδ T cell-derived M-CSF. Those myeloid cells contribute significantly to the multifaceted immune response against *Plasmodium* parasites (63, 64). However, the recent study showed that γδ T cells only became the major source of M-CSF in the late phase of the infection (64).

The observed dysfunction of cDC1 in TCRδ KO mice resulted in impaired Th1 differentiation from *Plasmodium-*specific CD4^+^ “PbT-II” cells. Th1 differentiation was established from day 7 pi, with TCRδ KO mice exhibiting significantly lower proportions of CXCR6^+^CXCR5^–^ and Tbet^hi^TCF1^–^ PbT-II cells with a Th1 phenotype. This impairment was also reflected in the splenic distribution of PbT-II cells. We observed an increase in the proportion of PbT-II cells in the red pulp on day 7 pi in WT mice, where red pulp PbT-II cells mainly consist of Th1 cells (42). Conversely, no such increase was noted in TCRδ KO mice, consistent with impaired Th1 differentiation on day 7 pi. However, IFNγ^eYFP+^ PbT-II cells were detected as early as day 5 pi, with the major population being CXCR5^+^, suggesting a transitional Th1 differentiation status. These trans-Th1 cells were present in lower proportions and numbers in TCRδ KO mice, with a higher distribution in the splenic white pulp on day 5 pi. This distribution was reversed on day 7 pi, at which the majority of IFNγ^eYFP+^ fully differentiated Th1 cells could be found in the red pulp as expected. It is very likely that Th1 differentiation is initiated in the white pulp, where it is induced by the interaction of antigen-specific CD4^+^ T cells with mature cDC1 around day 5 pi and continues at later time points in the red pulp, influenced by other cell subsets including CXCL9-producing inflammatory monocytes (4). By contrast, the absence of γδ T cells did not impact the numbers of Tfh cells. It is possible that Tfh cells rely on cDC2 rather than on cDC1 for initial priming or that suboptimal priming by cDC1 is sufficient to initiate Tfh differentiation.

Finally, we investigated the requirement of cDCs for the activation of γδ T cells. Depletion of cDCs significantly impaired γδ T cell activation and function following infection. However, the exact mechanisms underlying this requirement remain unclear. Possible explanations include TCR-independent stimulation either by cytokines (e.g. IL-2, IL-12, IL-18) or costimulatory molecules (65–68). Another possibility is γδ TCR direct engagement with DCs. Human Vγ9^+^Vδ2^+^ γδ T cells respond to *Plasmodium falciparum* phosphoantigens through their TCR in MHC-independent manner (69). This recognition depends on butyrophilin family proteins BTN2A1 and BTN3A1. However, the phosphoantigen recognition was only observed in higher primates and alpacas, with no homologue found in mice (70). Previous study showed that murine Vγ1^+^Vδ6.3^+^ γδ T cell hybridomas can recognize bacterial and mammalian heat shock protein HSP60, with a conserved epitope as the ligands (71, 72). It is possible that Vγ1^+^Vδ6.3^+^ γδ T cells respond to the homologous *Plasmodium* HSP60 during the infection. The CD11c^DTR^ model, while widely used, has limitations in specificity for cDC depletion. CD11c expression is not exclusive to cDCs but is found in several non-cDC subsets (73). Consequently, the observed impairment in γδ T cell activation may not solely result from cDC depletion but could involve other CD11c-expressing cell populations. Thus, we should further investigate the other cell populations that might have some impacts on the γδ T cell activation in the early phase of *Plasmodium* infection. CD169^+^ macrophages emerge as another potential key player in the γδ T cell activation during *Plasmodium* infection. These macrophages have been shown to contribute to recruitment and activation of γδ T cells in protective immunity against *Staphylococcus aureus* skin infection. Furthermore, CD169^+^ macrophages can transfer antigens to cDC1 in spleen for cross-presentation and subsequent activation of CD8^+^ cytotoxic T cells (74), This suggests a possible collaborative interaction between splenic cDC1 and CD169^+^ macrophages in the early phase of *Plasmodium* infection.

Our study uncovered a cooperative interaction between γδ T cells and cDC1 for adequate Th1 cell commitment of *Plasmodium*-specific CD4^+^ T cells in the initial phase of malaria. However, further studies are warranted to ascertain whether a specific cDC subset is responsible for the activation of γδ T cells and the nature of γδTCR ligands involved in this activation. In this study, we did not confirm the direct cell-interaction by *in vitro* culture system. Thus, further study is important to elucidate the mechanism for reciprocal activation of γδ T cells and cDC1 for adequate Th1 cell-differentiation against *Plasmodium* infection. Considering the importance of cellular immunity in the protection and pathology onset of *Plasmodium* infection, the findings of our study could provide new insights into the optimal conditions for eliciting the desired Th1 response while avoiding immune pathology. This knowledge could have significant implications for the development of future malaria vaccines.

## Data Availability

The raw data supporting the conclusions of this article will be made available by the authors, without undue reservation.
