# Supplementary material for: γδ T cell-mediated activation of cDC1 orchestrates CD4+ Th1 cell priming in malaria
Source: Front Immunol. 2024 Aug 15;15:1426316. doi: 10.3389/fimmu.2024.1426316 (PMC11357926; doi:10.3389/fimmu.2024.1426316)
Supplement: Supplementary file 1 [file Table1.docx]

| **REAGENT or RESOURCE** | **SOURCE** | **IDENTIFIER** |
| --- | --- | --- |
| **Antibodies** | | |
| PE anti-human/mouse Bcl-6 Antibody | Biolegend | Cat# 358504 |
| CCL3 (MIP-1 alpha) Monoclonal Antibody (DNT3CC), PerCP-eFluor™ 710 | eBioscience | Cat# 46-7532-82 |
| PE anti-mouse CCL5 (RANTES) Antibody | Biolegend | Cat# 149103 |
| PE anti-mouse CD197 (CCR7) Antibody | Biolegend | Cat# 120105 |
| PE anti-mouse CXCL9 (MIG) Antibody | Biolegend | Cat# 515603 |
| FITC anti-mouse CD11a Antibody | Biolegend | Cat# 101106 |
| PE/Cyanine7 anti-mouse CD11a Antibody | Biolegend | Cat# 101122 |
| PE anti-mouse/human CD11b Antibody | Biolegend | Cat# 101208 |
| CD11b Monoclonal Antibody (M1/70), PE-Cyanine7 | eBioscience | Cat# 25-0112-82 |
| APC anti-mouse CD11c Antibody | Biolegend | Cat# 117310 |
| FITC Anti-Mouse CD11c | Tonbo Biosciences | Cat# 35-0114-U100 |
| PE anti-mouse CD169 (Siglec-1) Antibody | Biolegend | Cat# 142403 |
| APC/Cyanine7 anti-mouse CD19 Antibody | Biolegend | Cat# 115529 |
| CD19 Monoclonal Antibody (eBio1D3 (1D3)), PerCP-Cyanine5.5 | eBioscience | Cat# 45-0193-82 |
| APC anti-mouse CD19 Antibody | Biolegend | Cat# 115511 |
| FITC Anti-Mouse CD25 | Tonbo Biosciences | Cat# 35-0251-U100 |
| CD25 Monoclonal Antibody (PC61.5), PE | eBioscience | Cat# 12-0251-82 |
| PE/Cyanine7 Anti-Mouse CD25 | Tonbo Biosciences | Cat# 60-0251-U100 |
| APC/Cyanine7 anti-mouse CD3ε Antibody | Biolegend | Cat# 100330 |
| Brilliant Violet 510™ anti-mouse CD3ε Antibody | Biolegend | Cat# 100353 |
| Brilliant Violet 711™ anti-mouse CD3 Antibody | Biolegend | Cat# 100241 |
| PerCP anti-mouse CD3ε Antibody | Biolegend | Cat# 100325 |
| APC/Cyanine7 anti-mouse CD4 Antibody | Biolegend | Cat# 100414 |
| Brilliant Violet 711™ anti-mouse CD4 Antibody | Biolegend | Cat# 100447 |
| CD40 Monoclonal Antibody (HM40-3), FITC | eBioscience | Cat# 11-0402-82 |
| APC/Cyanine7 anti-mouse CD45.1 Antibody | Biolegend | Cat# 110716 |
| FITC anti-mouse CD45.1 Antibody | Biolegend | Cat# 110705 |
| PE anti-mouse CD45.1 Antibody | Biolegend | Cat# 110708 |
| Brilliant Violet 605™ anti-mouse CD45.2 Antibody | Biolegend | Cat# 109841 |
| FITC anti-mouse CD45.2 Antibody | Biolegend | Cat# 109806 |
| BV421 Rat Anti-Mouse CD49d | BD Bioscience | Cat# 740016 |
| FITC anti-mouse CD69 Antibody | Biolegend | Cat# 104505 |
| PE anti-mouse CD69 Antibody | Biolegend | Cat# 104508 |
| CD8a Monoclonal Antibody (53-6.7), eFluor™ 450 | eBioscience | Cat# 48-0081-82 |
| FITC Anti-Mouse CD8a | Tonbo Biosciences | Cat# 35-0081-U500 |
| FITC anti-mouse CD80 Antibody | Biolegend | Cat# 104705 |
| FITC anti-mouse CD86 Antibody | Biolegend | Cat# 105109 |
| Brilliant Violet 650™ anti-mouse CD183 (CXCR3) Antibody | Biolegend | Cat# 126531 |
| APC anti-mouse CD185 (CXCR5) Antibody | Biolegend | Cat# 145506 |
| PE anti-mouse CD186 (CXCR6) Antibody | Biolegend | Cat# 151103 |
| Alexa Fluor® 488 anti-mouse IFN-γ Antibody | Biolegend | Cat# 505813 |
| APC anti-mouse IFN-γ Antibody | Biolegend | Cat# 505810 |
| PE Rat Anti-Mouse IL-12 (p40/p70) | BD Bioscience | Cat# 554479 |
| APC anti-mouse CD223 (LAG-3) Antibody | Biolegend | Cat# 125210 |
| MHC Class II (I-A/I-E) Monoclonal Antibody (M5/114.15.2), FITC | eBioscience | Cat# 11-5321-85 |
| PE/Cyanine7 anti-mouse I-A/I-E Antibody | Biolegend | Cat# 107630 |
| CD314 (NKG2D) Monoclonal Antibody (CX5), PE | eBioscience | Cat# 12-5882-81 |
| FITC anti-mouse CD279 (PD-1) Antibody | Biolegend | Cat# 135213 |
| PE/Cyanine7 anti-mouse CD279 (PD-1) Antibody | Biolegend | Cat# 135215 |
| Brilliant Violet 421™ anti-mouse CD279 (PD-1) Antibody | Biolegend | Cat# 135221 |
| PE/Cyanine7 anti-T-bet Antibody | Biolegend | Cat# 644824 |
| PE Mouse Anti-TCF-7/TCF-1 | BD Bioscience | Cat# 564217 |
| BV510 Hamster Anti-Mouse TCR β Chain | BD Bioscience | Cat# 563221 |
| Brilliant Violet 421™ anti-mouse TCR β chain Antibody | Biolegend | Cat# 109230 |
| APC Anti-mouse TCR γ/δ Antibody | Biolegend | Cat# 118116 |
| TCR gamma/delta Monoclonal Antibody (eBioGL3 (GL-3, GL3)), PE | eBioscience | Cat# 12-5711-82 |
| PE/Cyanine7 anti-mouse TCR γ/δ Antibody | Biolegend | Cat# 118124 |
| PerCP/Cyanine5.5 anti-mouse TNF-α Antibody | Biolegend | Cat# 506322 |
| BV510 Hamster Anti-Mouse Vδ 6.3/2 TCR | BD Bioscience | Cat# 744473 |
| PE anti-mouse TCR Vγ1.1/Cr4 Antibody | Biolegend | Cat# 141106 |
| APC anti-mouse TCR Vγ2 Antibody | Biolegend | Cat# 137708 |
| PE/Cyanine7 anti-mouse NK-1.1 Antibody | Biolegend | Cat# 108713 |
| Pacific Blue^TM^ anti-mouse F4/80 Antibody | Biolegend | Cat# 123124 |
| Purified anti-mouse CD16/32 Antibody | Biolegend | Cat# 101302 |
| Purified anti-GFP Antibody | Biolegend | Cat# 338002 |
| DyLight™ 488 Goat anti-rat IgG Antibody | Biolegend | Cat# 405310 |
| **Chemicals, peptides, and recombinant proteins** | | |
| Phorbol 12-myristate 13-acetate (PMA) | Cayan Chemical Company | Cat# 10008014 |
| Brefeldin A | Cayan Chemical Company | Cat# 11861 |
| Ionomycin | Cayan Chemical Company | Cat# 10004974 |
| Diphtheria toxin | Bio Academia | Cat# 01-517 |
| PbT-II Peptide | SIGMA Genosys | Custom order |
| Block one histo | Nacalai tesque | Cat# 06349-64 |
| Fluorescent mounting medium | Agilent | Cat# S3023 |
| 7AAD | Cayan Chemical Company | Cat # 11397 |
| Dorbene🄬 vet | Kyoritsu Seiyaku Corporation |  |
| Midazolam Sandoz🄬 | Sandoz | Cat# 614243022 |
| Vetorphale🄬 | Meiji Seika Pharma |  |
| **Critical commercial assays** | | |
| BD cytofix/cytoperm kit | BD biosciences | Cat# 554714 |
| eBioscience™ Transcription Factor Fixation/Permeabilization kit | Invitrogen™ | Cat# 00-5521-00 |
| BD IMag™ Anti-Mouse CD4 Magnetic Particles - DM | BD biosciences | Cat# 551539 |
| CD11c MicroBeads UltraPure, mouse | Miltenyi Biotec | Cat# 130-125-835 |
| **Experimental models: Organisms/strains** | | |
| Mouse: TCRδ KO | RIKEN BioResource Research Center | BRC No. RBRC00407 |
| Mouse: PbT-II | William R Heath (University of Melbourne) | Fernandez-Ruiz et al ([2017](https://www.embopress.org/doi/full/10.15252/emmm.202317713#emmm202317713-bib-0012)) |
| Mouse: IFN-γ^eYFP^ | The Jackson Laboratory | Strain# 017580 |
| Mouse: PbT-II/IFN-γ^eYFP^ | This paper |  |
| Mouse: CD11c^DTR^ | The Jackson Laboratory | Strain# 004509 |
| *Plasmodium* *chabaudi* | Richard Culleton (Ehime University) |  |
| *Plasmodium* *berghei* ANKA | Masao Yuda (Mie University) |  |
| **Software and algorithms** | | |
| Adobe Illustrator 16.0.4 | Adobe | <https://www.adobe.com/adobe/illustrator> |
| FlowJo 10.9.0 | Treestar | <https://www.flowjo.com/> |
| GraphPad PRISM 8.0.0 | Graphpad software | [https://www.graphpad.com](https://www.graphpad.com/) |
| ImageJ2 2.14.0/1.54f | ImageJ | https://imagej.net/ij/ |
