# Supplementary material for: γδ T cell-mediated activation of cDC1 orchestrates CD4+ Th1 cell priming in malaria
Source: Front Immunol. 2024 Aug 15;15:1426316. doi: 10.3389/fimmu.2024.1426316 (PMC11357926; doi:10.3389/fimmu.2024.1426316)
Supplement: Supplementary file 2 [file Presentation1.pdf]

Supplementary Figures

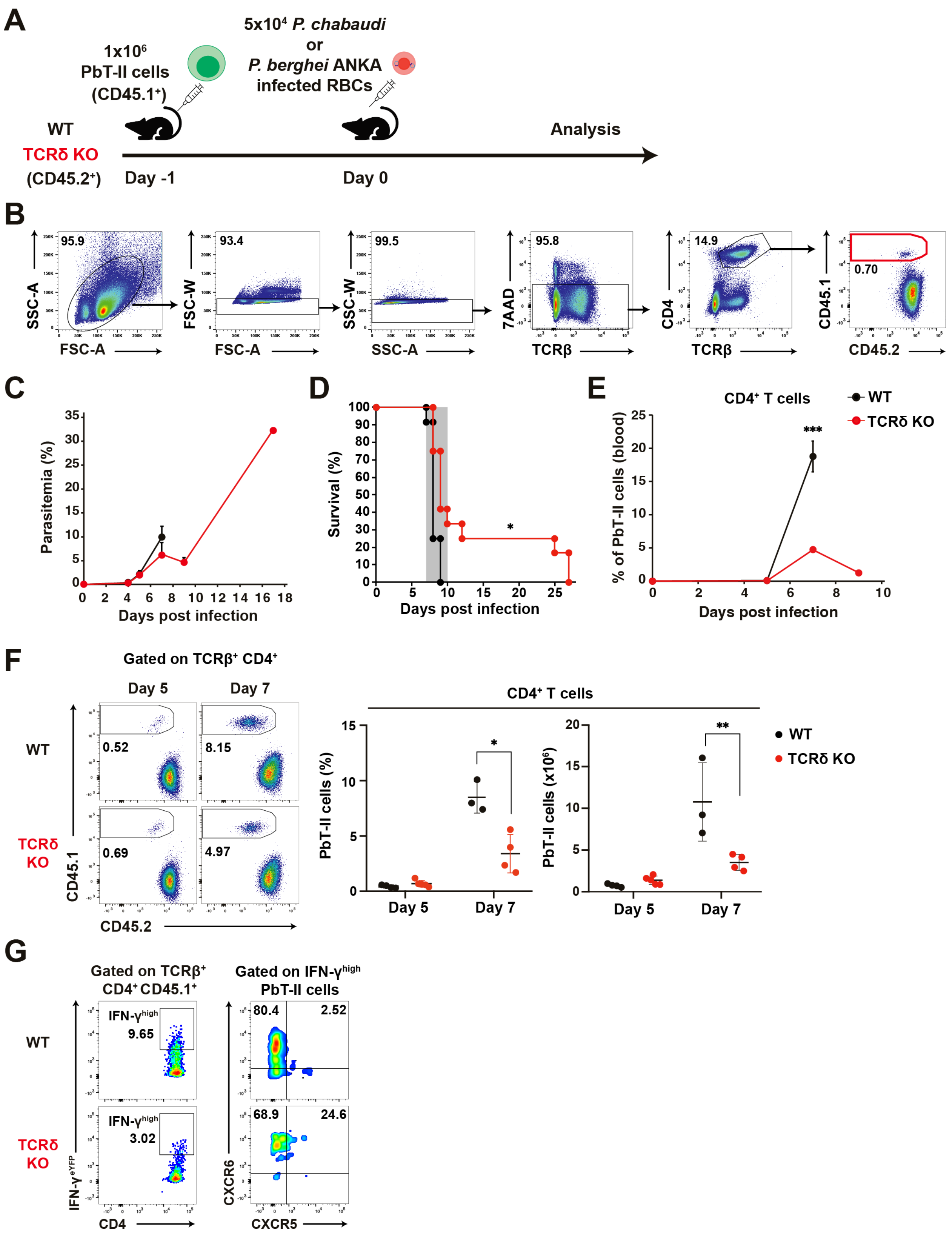

Figure S1

γδ T cells promote the response of *Plasmodium*-specific CD4$^+$ T cells in the acute phase of *P. berghei* infection (related to Figure 1)

(A) Experiment design.

(B) Gating strategy for PbT-II cells.

(C–F) PbT-II cells were transferred into WT and TCRδ KO mice 1 day prior to infection with *P. berghei* ANKA. Parasitemia **(C)**, survival curve **(D)**, and PbT-II cells **(E)** were monitored in peripheral blood. Gray area in **(D)** indicates development of experimental cerebral malaria.

(F) Representative flow plot (left), proportion (center), and number (right) of PbT-II cells (CD45.1$^+$) in the spleen.

(G) IFN-γ^{eYFP} PbT-II cells were transferred into WT and TCRδ KO mice 1 day prior to infection with *P. chabaudi*. Representative flow plot of eYFP^{high} in IFN-γ^{eYFP} PbT-II cells (left) and CXCR6$^+$ in eYFP^{high} PbT-II cells (right) on day 7pi.

∗p<0.05; ∗∗p<0.01; ∗∗∗p<0.001; Student’s t-test. Each symbol represents an individual mouse, while error bars indicate the SD, Log-rank test was used in **(D)**. **(C, E, F, G)** Data plots were representative one of two experiments, 3–5 mice per group. **(D)** data were pooled from 6 mice per group from two experiments.

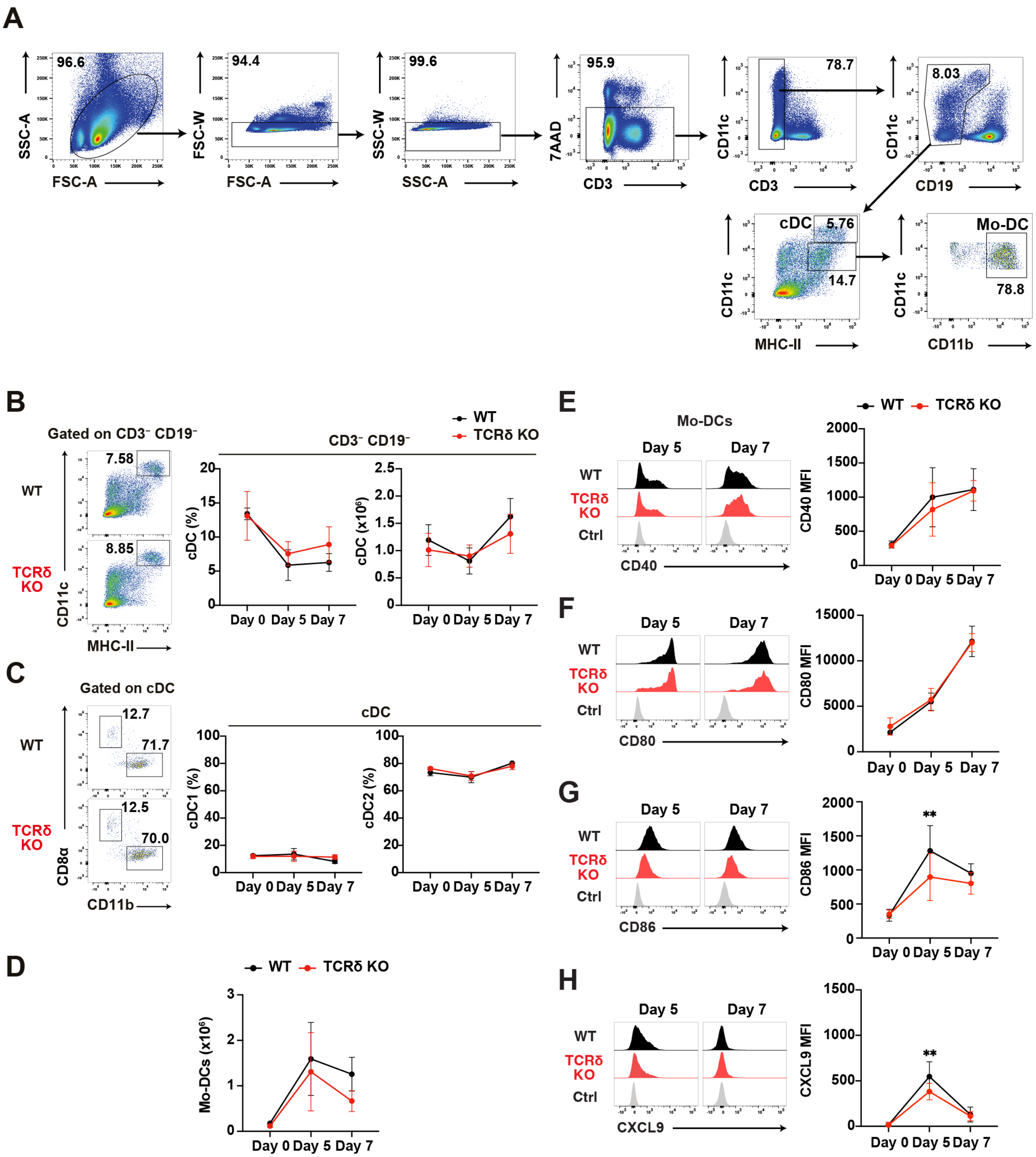
A
SSC-A
FSC-A
96.6
FSC-W
94.4
SSC-W
99.6
7AAD
CD3
95.9
CD11c
78.7
CD19
8.03
cDC
5.76
14.7
MHC-II
Mo-DC
78.8
CD11b
B
Gated on CD3⁻ CD19⁻
WT
TCRδ KO
7.58
8.85
CD3⁻ CD19⁻
cDC (%)
cDC (x10⁶)
Day 0 Day 5 Day 7
C
Gated on cDC
12.7
71.7
12.5
70.0
CD8α
cDC
cDC1 (%)
cDC2 (%)
D
Mo-DCs (x10⁶)
E
Mo-DCs
Day 5
Day 7
Ctrl
CD40
CD40 MFI
F
CD80
CD80 MFI
G
CD86
CD86 MFI
**
H
CXCL9
CXCL9 MFI
**

Figure S2

Impact of γδ T cells on dendritic cell numbers during malaria infection (related to Figure 3)

(A) Gating strategy for cDCs and Mo-DCs.

(B) Representative flow plots (left), proportions (center), and numbers (right) of splenic cDCs. **(C)** Representative flow plots (left), proportion of cDC1 (center), and cDC2 proportions (right) within splenic cDC.

(D) Number of splenic Mo-DCs.

(E–H) Representative histogram (left) and average MFI (right) of CD40 **(E)**, CD80 **(F)**, CD86 **(G),** and CXCL9 **(H)** in splenic Mo-DCs.

$*p<0.05$; $**p<0.01$; $***p<0.001$; Student's t-test. Each symbol represents an individual mouse, while error bars indicate the SD. **(B–G)** Data were pooled from 3 mice per group (day 0) from one experiment, 7–13 mice per group (day 5) from four experiments, and 6 mice per group (day 7) from two experiments. **(H)** Data were pooled from 3 mice per group (day 0) from one experiment and 8 mice per group (day 5) from two experiments and 5 mice per group (day 7) from one experiment. "Ctrl" represents isotype control.

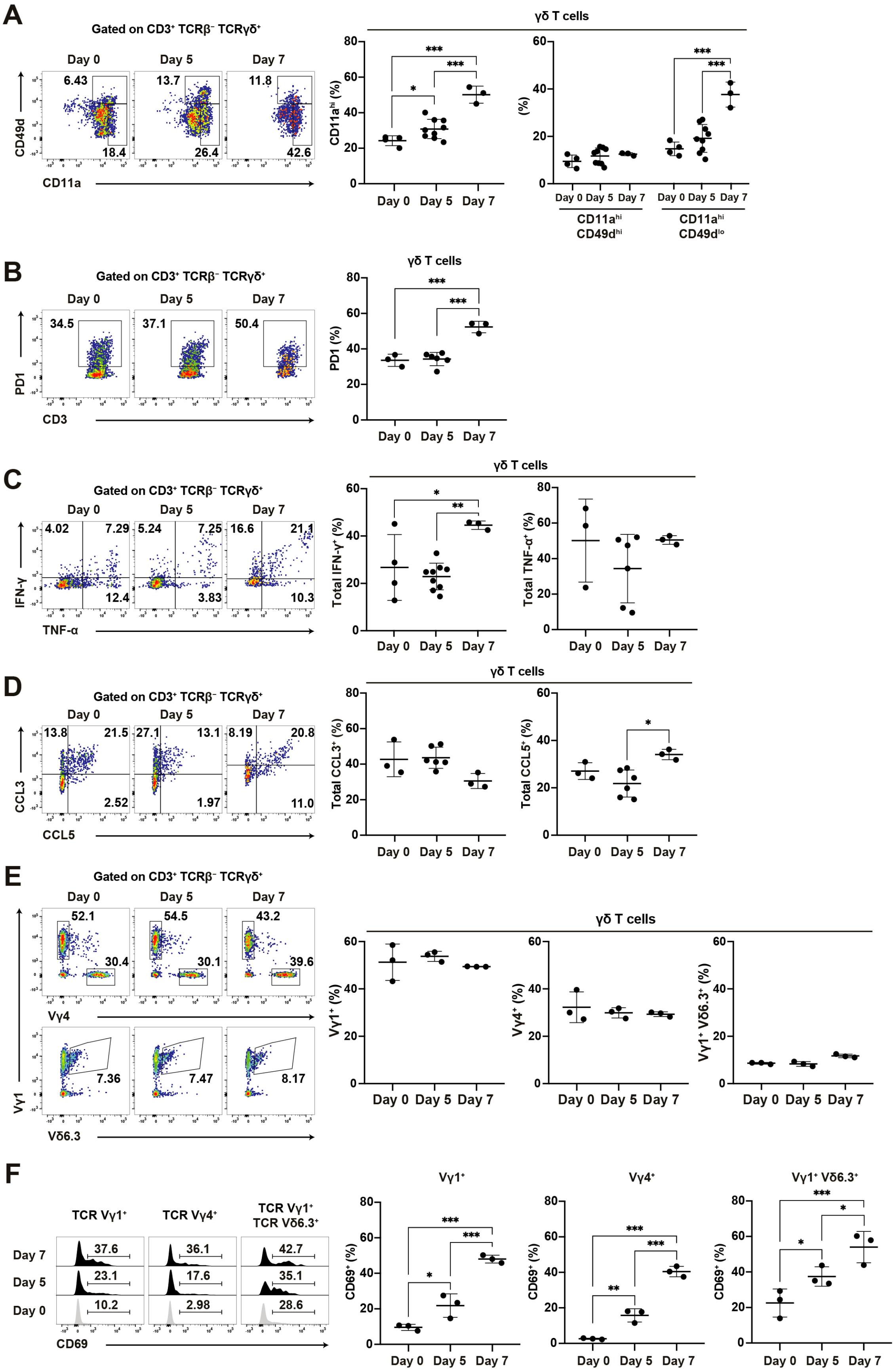
A
Gated on CD3⁺ TCRβ⁻ TCRγδ⁺
Day 0
Day 5
Day 7
6.43
18.4
13.7
26.4
11.8
42.6
CD49d
CD11a
γδ T cells
CD11a^hi (%)
Day 0 Day 5 Day 7
CD11a^hi CD49d^hi
CD11a^hi CD49d^lo
B
Gated on CD3⁺ TCRβ⁻ TCRγδ⁺
34.5
37.1
50.4
PD1
CD3
γδ T cells
PD1 (%)
C
Gated on CD3⁺ TCRβ⁻ TCRγδ⁺
4.02
7.29
12.4
5.24
7.25
3.83
16.6
21.1
10.3
IFN-γ
TNF-α
γδ T cells
Total IFN-γ⁺ (%)
Total TNF-α⁺ (%)
D
Gated on CD3⁺ TCRβ⁻ TCRγδ⁺
13.8
21.5
2.52
27.1
13.1
1.97
8.19
20.8
11.0
CCL3
CCL5
γδ T cells
Total CCL3⁺ (%)
Total CCL5⁺ (%)
E
Gated on CD3⁺ TCRβ⁻ TCRγδ⁺
52.1
30.4
54.5
30.1
43.2
39.6
Vγ4
7.36
7.47
8.17
Vγ1
Vδ6.3
γδ T cells
Vγ1⁺ (%)
Vγ4⁺ (%)
Vγ1⁺ Vδ6.3⁺ (%)
F
TCR Vγ1⁺
TCR Vγ4⁺
TCR Vγ1⁺ TCR Vδ6.3⁺
Day 7
Day 5
Day 0
37.6
23.1
10.2
36.1
17.6
2.98
42.7
35.1
28.6
CD69
Vγ1⁺
Vγ4⁺
Vγ1⁺ Vδ6.3⁺
CD69⁺ (%)

Figure S3

Activation of γδ T cells across multiple subsets during the initial phase of malaria (related to Figure 4)

(A) Representative flow plots (left), proportion of $CD11a^{hi}$ (center), and proportion of $CD11a^{hi}$ $CD49d^{hi/lo}$ (right) in splenic γδ T cells.

(B) Representative flow plots (left) and proportion (right) of $PD1^{+}$ γδ T cells.

(C) Representative flow plots (left), proportion of IFN-γ^{+} (center), and proportion of $TNF\alpha^{+}$ (right) in splenic γδ T cells following PMA/Ionomycin stimulation.

(D) Representative flow plots (left), proportion of $CCL3^{+}$ (center), and proportion of $CCL5^{+}$ (right) in splenic γδ T cells following PMA/Ionomycin stimulation. **(A–D)** Data pooled from 3–9 mice per group from four experiments.

(E) Representative flow plots (left) and proportion of $V\gamma1^{+}$, $V\gamma4^{+}$, and $V\gamma1^{+}V\delta6.3^{+}$ in splenic γδ T cells.

(F) Representative histograms (left) and average MFI of CD69 within $V\gamma1^{+}$, $V\gamma4^{+}$, and $V\gamma1^{+}V\delta6.3^{+}$ subsets. **(E and F)** Data pooled from 3 mice per group from three experiments. ∗$q<0.05$; ∗∗$q<0.01$; ∗∗∗$q<0.001$; One-way ANOVA. Each symbol represents an individual mouse, while error bars indicate the SD.

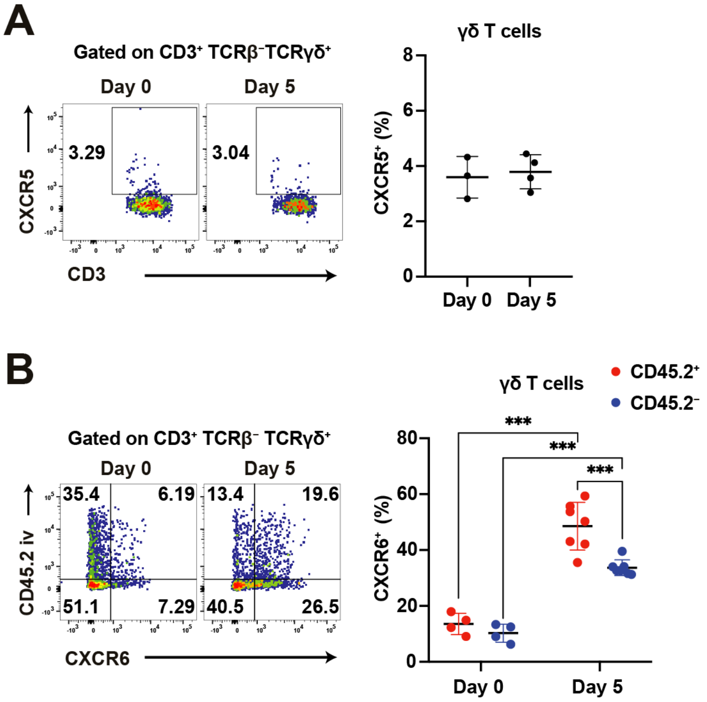

Figure S4

γδ T cell accumulation in the splenic white pulp with no significant association with CXCR5/CXCR6 expression (related to Figure 6)

(A) Representative flow plots (left) and proportions (right) of $CXCR5^+$ γδ T cells. Data pooled from 3–4 mice per group from two experiments.

(B) Representative flow plots (left) and proportion (center) of $CXCR6^+$ cells in splenic γδ T cells from *in vivo* CD45.2-labeled WT mice. Red dots represent the proportion in $CD45.2^+$ γδ T cells, while blue dots represent the proportion in $CD45.2^-$ γδ T cells. Data pooled from 3–7 mice per group from three experiments. Student's t-test was used. p<0.05; **p<0.01; ***p<0.001.

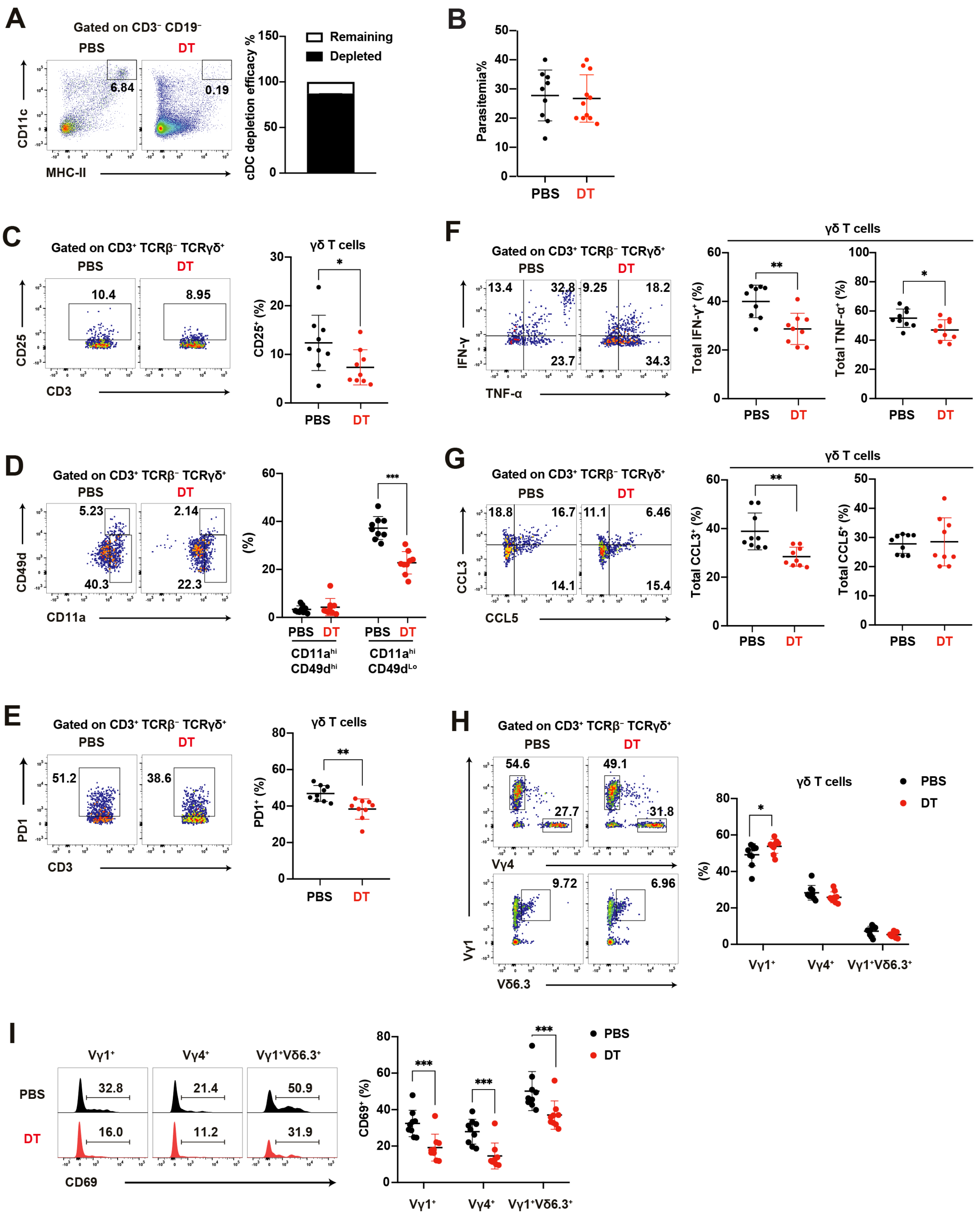

A
Gated on CD3⁻ CD19⁻
PBS
DT
6.84
0.19
CD11c
MHC-II
cDC depletion efficacy %
Remaining
Depleted
B
Parasitemia%
PBS
DT
C
Gated on CD3⁺ TCRβ⁻ TCRγδ⁺
10.4
8.95
CD25
CD3
γδ T cells
CD25⁺ (%)
D
5.23
2.14
40.3
22.3
CD49d
CD11a
(%)
CD11a^hi CD49d^hi
CD11a^hi CD49d^Lo
E
51.2
38.6
PD1
PD1⁺ (%)
F
13.4
32.8
9.25
18.2
23.7
34.3
IFN-γ
TNF-α
Total IFN-γ⁺ (%)
Total TNF-α⁺ (%)
G
18.8
16.7
11.1
6.46
14.1
15.4
CCL3
CCL5
Total CCL3⁺ (%)
Total CCL5⁺ (%)
H
54.6
27.7
49.1
31.8
9.72
6.96
Vγ4
Vγ1
Vδ6.3
Vγ1⁺
Vγ4⁺
Vγ1⁺Vδ6.3⁺
I
32.8
21.4
50.9
16.0
11.2
31.9
CD69
CD69⁺ (%)

Figure S5

Depletion of cDCs suppresses γδ T cell activation across multiple subsets (related to Figure 6)

(A) Representative flow plots (left) and summary (right) of the efficacy of DT-mediated depletion of cDCs in chimera mice on day 7 pi.

(B) Parasitemia on day 7 pi.

(C) Representative flow plots (left) and proportion (right) of $CD25^{+}$ splenic γδ T cells on day 7 pi.

(D) Representative flow plots (left) and proportion (right) of $CD11a^{hi}$ $CD49d^{hi/lo}$ splenic γδ T cells on day 7 pi.

(E) Representative flow plots (left) and proportion (right) of $PD1^{+}$ in splenic γδ T cells on day 7 pi.

(F) Representative flow plots (left), proportion of total IFN-γ^{+} (center), and total TNFα^{+} (right) in γδ T cells following PMA/Ionomycin stimulation on day 7 pi.

(G) Representative flow plots (left), proportion of total $CCL3^{+}$ (center), and total $CCL5^{+}$ (right) in γδ T cells following PMA/Ionomycin stimulation on day 7 pi.

(H) Representative flow plots (upper) and proportions (lower) of $V\gamma1^{+}$, $V\gamma4^{+}$, and $V\gamma1^{+}V\delta6.3^{+}$ subsets within splenic γδ T cells on day 7 pi.

(I) Representative histograms (left) and proportions (right) of $CD69^{+}$ within $V\gamma1^{+}$, $V\gamma4^{+}$, and $V\gamma1^{+}V\delta6.3^{+}$ subsets on day 7 pi. ∗$p<0.05$; ∗∗$p<0.01$; ∗∗∗$p<0.001$; Student's t-test. Each symbol represents an individual mouse, while error bars indicate the SD. Data pooled from 9 mice per group from three experiments.

A Steady state

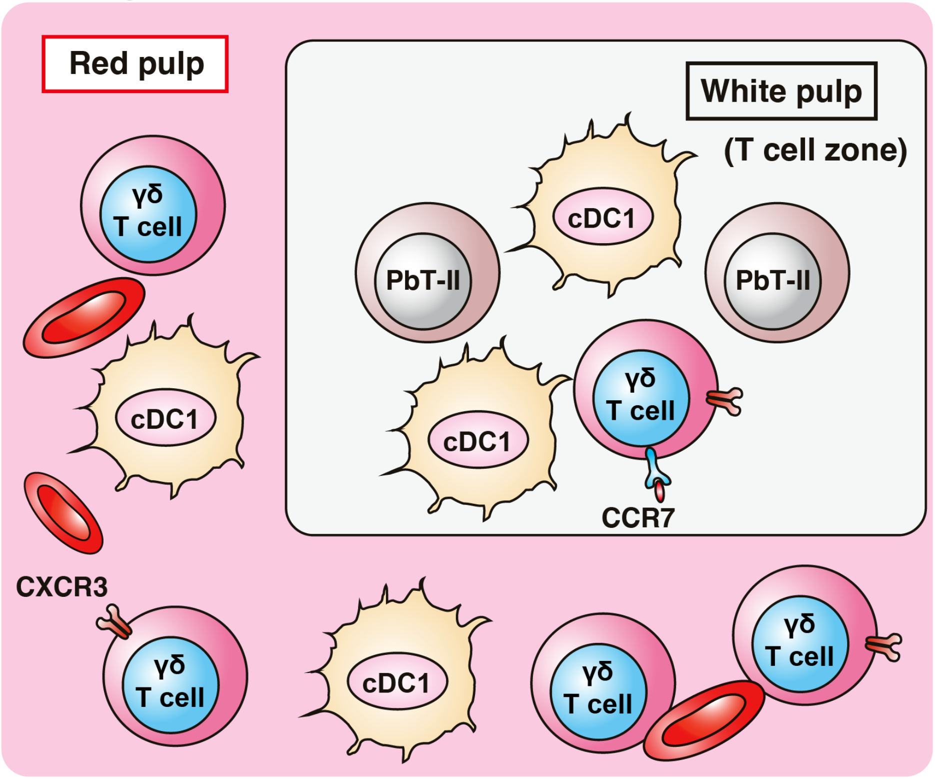

B Early phase *Plasmodium* infection

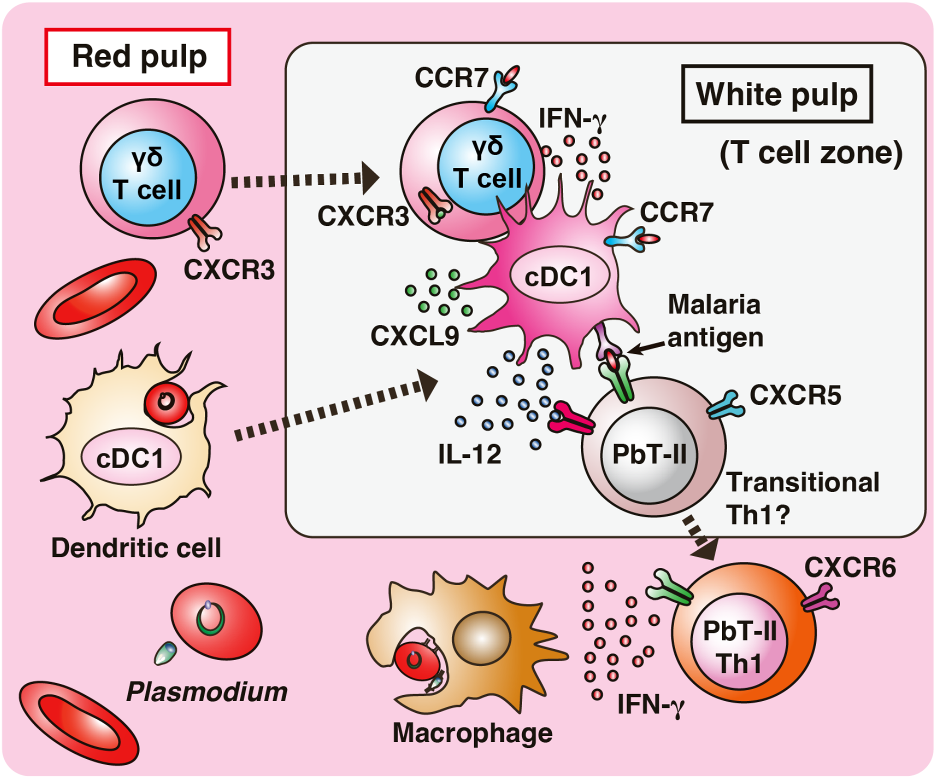

Figure S6

Changes in the immune landscape in the spleen during *Plasmodium* infection

(A) Splenic distribution of cDC1, γδ T cells, and PbT-II cells at steady state. cDC1 were evenly distributed between the T cell zone in the white pulp and the red pulp. PbT-II cells were primarily located in the T cell zone within the white pulp. γδ T cells had a relatively higher presence in the red pulp. CCR7 expression on γδ T cells mediated their localization to the white pulp. By contrast, CXCR3 was expressed by a subset of γδ T cells regardless of their localization.

(B) The immune landscape of the spleen in the early phase of *Plasmodium* infection. CCR7 upregulation in cDC1 drives their relocation from the red pulp to the T cell zone in the white pulp. γδ T cells also accumulated in the white pulp, although this did not coincide with CCR7 upregulation. Furthermore, cDC1/γδ T cell interactions increased in the early phase of infection. These interactions were dependent on CXCR3 signaling in γδ T cells. As a result, cDC1 achieved optimal maturation and increased IL-12 production, allowing them to prime PbT-II cells to initiate Th1 differentiation. At this time point, activated PbT-II cells were still undergoing a Th1/Tfh transitional state. A subset of these CXCR5-expressing PbT-II cells produced IFN-γ, referred to as "trans-Th1." These trans Th1 cells were mainly found within the white pulp in the early phase of the infection, unlike fully differentiated Th1 cells observed mainly in the red pulp at later time points.
